# Willingness to sacrifice among convicted Islamist terrorists versus violent gang members and other criminals

**DOI:** 10.1038/s41598-022-06590-0

**Published:** 2022-02-16

**Authors:** Angel Gómez, Scott Atran, Juana Chinchilla, Alexandra Vázquez, Lucia López-Rodríguez, Borja Paredes, Mercedes Martínez, Laura Blanco, Beatriz Alba, Hend Bautista, Saulo Fernández, Florencia Pozuelo-Rubio, José Luis González-Álvarez, Sandra Chiclana, Héctor Valladares-Narganes, María Alonso, Alfredo Ruíz-Alvarado, José Luis López-Novo, Richard Davis

**Affiliations:** 1 Artis International, Scottsdale, AZ 85254 USA; 2 Facultad de Psicología, Universidad Nacional de Educación a Distancia, 28040 Madrid, Spain; 3 Changing Character of War Centre, University of Oxford, Oxford, OX1 1DW UK; 4 Gerald Ford School of Public Policy, University of Michigan, Michigan, 48109 USA; 5 Departamento de Psicología, Universidad de Almería, 04120 Almería, Spain; 6 Facultad de Psicología, Universidad Autónoma de Madrid, 28049 Madrid, Spain; 7 Instituciones Penitenciarias, Ministerio del Interior, Gobierno de España, 28014 Madrid, Spain; 8 Secretaría de Estado de Seguridad, Ministerio del Interior, Gobierno de España, 28010 Madrid, Spain; 9 School of Politics and Global Studies, Arizona State University, Tempe, 873902 USA

**Keywords:** Psychology, Human behaviour

## Abstract

Is terrorism just another form of criminal activity, as many nations’ justice systems assume? We offer an initial answer using face-to-face interviews and structured surveys in thirty-five Spanish prisons. Recent theories of extreme sacrifice inform this direct observational and comparative study. Islamist terrorists display levels of self-sacrifice for their primary reference group similar to that of Latino gangs, but greater willingness to sacrifice for primary values than other inmates (non-radical Muslims, Latino gangs, and delinquent bands). This disposition is motivated by stronger perceived injustice, discrimination, and a visceral commitment to such values (risk/radicalization factors). Nevertheless, state authorities, prison staff, and families are (protective/de-radicalization) factors apt to reduce willingness to sacrifice and keep foreign fighters, now being released in large numbers, from returning to terrorism.

## Introduction

Despite global reduction in Islamist terrorist attacks since territorial defeat of the Islamic State of Iraq and Syria (ISIS or ISIL)^1^, serious transnational threats remain as terrorist forces regroup while governments are preoccupied with managing a global pandemic^2^. Despite the rapid rise of far-right and racial supremacist violence, Islamist terrorism remains a serious threat, with many international security services echoing this UN Security Council warning:Other threats from ISIL, Al-Qaida and their ideology continue to challenge Governments and security forces [with] issues related to … effective prosecution of returned foreign fighters, prison radicalization and a wave of pending releases, especially from prisons in Europe.”^3^ In 2020 alone, an estimated 1000 such prisoners were scheduled for release. The recent Taliban victory over United States, NATO and Afghan government forces in Afghanistan has also raised worldwide concern about a possible resurgence of global Islamist violence^4^.

Many governments^5^ and international bodies^6^ treat terrorists, particularly Islamist terrorists, like members of other criminal associations^7^. Although the interest in studying violent extremism and radicalization is a global ambition, in addition to difficulty accessing samples of terrorists, gaps in the current research literature arguably require more focused investigation, particularly regarding the characterization of terrorist violence as criminal behavior. For example: (1) although research suggests that terrorist offenders can have much in common with criminal offenders when it comes to risk factors^8^, there may be a significant overestimation of the similarity between Islamist terrorism and gang behavior; (2) empirical data is still seriously lacking in regard to claims about the similarities and differences between jihadist and non-jihadist criminal groups in terms of the psychosocial factors that motivate their respective behaviors^8,9^, and particularly what may affect recidivism^10,11^; (3) there has been scant attempt to undertake direct comparative field research, or to coordinate and synthesize qualitative and quantitative data; and (4) the comparative role played by psychosocial factors has been under-researched^9^. What follows is intended to help fill these gaps.

First, Islamist terrorist groups are often compared to criminal groups, including informal criminal street bands, ritually-marked violent transnational gangs (e.g., MS-13), and hierarchically-structured crime syndicates (e.g., Camorra)^12^. Some comparisons note that gangs and crime syndicates may also seek political power and social influence (e.g., “narco-terrorists”), forge alliances with terrorist groups, and recruit from the same marginalized populations^13,14^. As one study put it: “ISIS’s violence and criminality are… readily understood through the lens of street gangs.”^15^ Others argue that some inflate similarities between gangs and terrorists for the added attention and assistance that claims of “terrorism” can bring, when in fact: “Mara [MS-13] is not really a transnational organization [and] its relationship with Al Qaeda and terrorism is overblown.”^16^.

Criminological research on individual gang members^17^, reveals that “a personal code of honor” with a “value system”^18^, regulates conduct as right or wrong, strengthens solidarity, preserves discipline, and protects members from enemies. As with some criminal organizations like the Italian Mafia^19^, collectively held ideological beliefs related to personal honor and masculinity in Latino gangs tend to favor violence when “personal space” is violated so that “derogation of one of their members affects their collective honor”^20^. In contrast to jihadist groups, however, such value systems regarding the honorableness of violence when norms of interpersonal etiquette are violated are frequently related to issues of material profit or interest and not primarily connected to the ideological beliefs of a broad societal movement, much less national or global revolution^21^.

As evident from field studies and experiments, Islamist terrorists are often revolutionary insurgents and dedicated combatants^22^, with absolute ideological commitments to religious, political, or cultural values that can motivate costly sacrifices and extreme violence^23^. They not only have those values, but also want to convert others to them. Transnational Islamist groups, especially, act to radically change values for the whole of society and even the world, and to greatly expand membership into non-criminal, mainstream populations^7^. Strategies developed for criminal gangs do not address these issues. In particular, we anticipate that: Islamist terrorists are more committed to their values than gang members and other criminal offenders; these shared values are critical for the enduring relationship that jihadists establish with their group; and jihadists are more willing to engage in extreme self-sacrifices for their values. Our study examines these anticipated differences between imprisoned Islamist terrorists and other prisoners, along dimensions than may inform counter-terrorism policy, and help impede Islamist terrorists’ movements from growing. Although the present study cannot directly support claims about other sorts of terrorist groups, such as those belonging to the rapidly rising tide of far-right and supremacist extremism, recent comparative analyses of jihadist and far-right terrorism intimate that findings from the present study may profitably inform comparative research on other forms of terrorism in relation to criminality^14,24–26^.

Second, there are strong theoretical and methodological reasons to assume that the systematic comparison of convicted terrorists with other criminal offenders may help to gain a deeper understanding of the nature of willingness to sacrifice among Islamist terrorists. Inasmuch as there is already considerable research and understanding into criminal offenders, and at least some significant commonalities with terrorists regarding risk factors^8^, we may be able to learn more about terrorist offenders by comparing them to criminals than to the general population. Although researchers have compared terrorists with gang members and ordinary criminals over the years^17,21,27–32^, there is a lack of comparative studies based on direct interaction with participants from these groups in a common environmental setting. Based on previous fieldwork with terrorists and other violent groups^22,26^, we operate on the assumption that direct comparisons of terrorist with other criminal groups in real-world settings are likely to have greater ecological validity using field-tested quasi-experimental designs than lab experiments that are often designed for controlled university student settings.

In brief, the present study attempts to overcome these two first limitations (i.e., overestimating confluence of terrorists and gangs, lack of direct comparative data) by conducting a quasi-experimental investigation of Islamist terrorists and gang members living in the same prison conditions, and by using the same methodology.

Third, whereas research on causes and motivations of terrorism is still relatively young and its findings somewhat controversial and inconclusive^33^, especially in regard to prison and post-prison behavior^34^, the longer tradition in the study of violent gangs, their risk factors and prison behaviors is relevant to the study of terrorism, radicalization and extremism by providing a comparative basis for such study^28^. We contend that such comparative study is well served via theories that can be reliably tested through triangulation of methods (i.e., qualitative and quantitative) in real-world field conditions, despite the evident challenges of research in such conditions (e.g., difficulties obtaining cooperation of authorities as well as inmates to unmonitored interviews in a secure environment).

Fourth, previous research indicates that socio-economic and psychosocial factors are critical to understanding likely involvement in organized crime and terrorism, although the psychosocial factors are arguably under-researched relative to socio-economic factors^9^. Moreover, whereas criminals and terrorists often may stem from the same general pool of subjects, they may well differ in how psychosocial factors influence their respective behaviors. Here, we provide theoretical support for determining key variables that predict willingness to make costly sacrifices, as well as those factors that risk enhancing or are liable to protect against such willingness.

Our conceptual frame is that of Devoted Actors^35^ experiencing Identity Fusion^36^, combining individual- and group-level theories. The Devoted Actor framework explains extreme value-driven behavior, including willingness to fight and die for a cause, regardless of material risks, costs, rewards, or consequences^37^. Identity Fusion involves an inviolable sense of oneness with a group, merging of individual with collective identity to predict extreme pro-group behavior and self-sacrifice^38^. The choice of identity fusion for our theoretical framework is inspired by three main findings from previous research: (1) the effects of fusion as predictor of extreme behaviors have been consistently tested as an extraordinary drive that leads individuals to risk their life and personal well-being for a group or for a conviction^39^; (2) fusion has been extensively validated in diverse cultural settings^40^; and (3) a recent meta-analyses^8^ indicates that fusion is a top risk factor predicting radical intentions. Similarly for cherished values^41^; behavior and brain studies in diverse cultural settings reveal that such values, which are dissociated from material tradeoffs, are highly predictive of willingness to make costly sacrifices^23^, including willingness to fight and die^42^; and they prove to be a key component of radical and polarized worldviews that lead to extreme violence^2^. Although research shows identity fusion and materially dissociated values to be somewhat independent predictors of extreme attitudes and violent behaviors, their interaction appears to maximize their effect on willingness to make costly sacrifices^43,44^.

Previous online studies indicate that for fused individuals being excluded and rejected increased endorsement of extreme pro-group actions to reaffirm social identity as a group member^45^. For Muslim fighters on the battlefield in Libya, identity fusion with comrades-in-arms is a strong predictor of willingness to fight and die^46^. For Muslims in Europe who feel marginalized and continue to face discrimination, support grows for radical groups^47^, seen to promote violence in the community’s defense^45^. Among frontline fighters in Iraq -captured ISIS, Marxist-Leninist PKK (considered a terrorist organization by the U.S. and Turkey), Kurdish Peshmerga, Iraqi army, and Sunni Arab militia- those who express greatest willingness to sacrifice (including fighting and dying) and in fact suffered the most battlefield casualties, displayed greater willingness to sacrifice for their most cherished values than even for primary reference group or family^22^. Behavioral and neuroimaging studies of radicalizing Muslim youth in Spain demonstrate that heightened perception of hostile and unjust social intent leads to “sacralization” of values one had not been previously willing to fight and die for^48^. Perceived discrimination and injustice have been also identified among the chief predictors of radical intentions^8^. Given these antecedents, we sought to also explore the role that perceptions of injustice and personal discrimination might play in mediating the relationships between fusion with groups, values, and willingness to sacrifice among prisoners.

In the case of protective factors, a recent review of prison programs across the European Union^49^, surmises that the most promising “rehabilitation interventions” involve: (1) individualized psychological engagement with prison personnel to reduce prisoner sentiments of personal discrimination and social marginalization, (2) prison staff training in recognition and respect for prisoners’ religious values and in discussing alternative perspectives for preventing violence, and (3) actively engaging families and friends as partners in the disengagement process. Increasing psychological engagement and perceived recognition of the prison staff may entail improvement of relational ties with the justice system in general, where the strength of relational ties can be measured by way of identity fusion^50^. Also, several studies suggest that the development of strong familial ties may contribute to the establishment of healthy relationships with other society members^8,51,52^, and thus be a potential driving force for the rehabilitation and reintegration of violent extremist offenders^53^. Noting that fostering family bonds based on care^54^ as well as kin-based sociocultural mores that are historically deep-seated^26^ constitute one of the most cherished values in the Muslim world, we also hypothesized that the degree of fusion with the justice system and with the family would act as protective factors against willingness to sacrifice among jihadists.

In sum, the present study endeavors to overcome some important limitations in prior studies that compare Islamist terrorists with different groups of criminals. It does this by using the Devoted Actor framework and a coordinated qualitative and quantitative methodology to directly compare convicted Islamist terrorists with Latino gang members, non-jihadist Muslim convicts, and non-Muslim Delinquent band members in the same prison environments. The focus is on the role of somewhat understudied psychosocial variables that go beyond socio-demographic and contextual factors. We offer a direct observational and structured empirical comparison, examining whether jihadists differ from gang members and other convicts in (1) their attachment to their primary reference groups’ members and values and (2) their willingness to sacrifice for them. We also ask what (3) factors favor and sustain radicalization in prison (e.g., feelings of injustice, personal discrimination) and (4) what mitigating factors might lessen willingness to sacrifice (e.g., identity fusion with family and with the nation’s justice system). Our results favor potentially potent programs for reducing and preventing radicalization and recidivism.

## The current study

The present study was carried out in Spanish prisons. Spain is distinguished by a large number of Islamist terrorist arrests^55^, being one of the European countries with the highest levels of Islamist radicalization, and the site of some of Europe’s worst terrorist attacks (e.g., the 2004 Madrid train bombings and the 2017 van and knife attacks in and around Barcelona^56^). Spain’s territorial foothold in Morocco (Ceuta and Melilla) and its close proximity to Muslim North Africa also makes it a favored site of illegal activity between Europe and North Africa, such as drug trafficking involving both Muslim and non-Muslim actors^57^. And Spain’s uniquely profound historical and linguistic connection with Latin America renders it a preferred European location for Latino-gangs^58^.

To conduct the study, the Spanish government was willing to give access to all prisons and prisoners in the penal system, under conditions conducive to candid observation. No one other than a member of our research team and a prisoner were present in the same room, even with those who were kept in isolation.

We conducted 350 in-depth, face-to-face interviews in 35 Spanish prisons (for prison distribution map, see Fig. S1). All participants were male and voluntary. Although there were few initial refusals, 73 participants were excluded from subsequent analyses because of mental health problems, refusal to answer most questions, or failure on a lie detection questionnaire. Participants acknowledged human subjects protections through informed consent (registered in Supplementary). Authorities had no access to individual prisoner responses.

We analyzed four classes of prisoners readily distinguished by the Spanish Ministry of Interior’s filing system: (a) Jihadists (Islamist terrorists, *N* = 57), including returning ISIS foreign fighters, participants in the 2004 Madrid train bombings (Europe’s deadliest terrorist attack), and in the lethal August 2017 vehicle attack on pedestrians in Barcelona; (b) Latino Gangs (*N* = 79) including murderers and violent offenders from Trinitarios and DDP (Dominican gangs founded in New York), Ñetas (Puerto Rican), Latin Kings (largest Hispanic street gang worldwide), and MS-13 (Mara Salvatrucha, founded in Los Angeles, arguably the most violent Latino gang); (c) Individual non-jihadists Muslims [unconnected with criminal organizations, (*N* = 94)]; and (d) Delinquent bands [local non-Muslim groups, (*N* = 47)]. The latter two groups mainly involved localized criminal activity (drug trafficking, robbery, assault and battery, etc.).

The study’s design had two stages during a single session, combining qualitative and quantitative analyses: namely, minimally-structured interviews followed by a questionnaire, lasting between 60 and 120 min. In the first stage, participants were asked to describe a normal day in their prison life, from awakening until day’s end. Next, they were asked to relate their life history from childhood to the present. In the second stage, the data were collected through a survey using the self-developed offline version of the magi-wise survey platform, which allows to combine traditional scales with dynamic measures. The critical measures were: (a) how they perceive their degree of fusion with their primary group in the past (just before entering into prison), and how they perceive their degree of fusion in the present; (b) factors enabling fusion with primary group (shared positive experiences, shared negative experiences, values); (c) fusion with primary value (also their perceived fusion in the past and present); (d) potential sacrifices for group and value; and (e) risk factors facilitating a positive relation between fusion and sacrifice (perceived injustice in sentencing, perceived personal discrimination), and protective factors favoring a negative relation (fusion with family, and Spain’s national justice system).

Based on the theoretical framework of Identity Fusion and the Devoted Actor, the focal categories for our initial measures—fusion (with primary group and value) and willingness to sacrifice (for the primary group and value)—were based on a preliminary study with a smaller sample (*N* = 52), where we asked: (1) “What is the most important group in your life, apart from your family, and why?” and (2) “What are the most important values in your life, and why?” (see supplementary online materials for details and examples). “Muslims” was almost always the primary group chosen by jihadists and Muslim non-jihadists, whereas the members of Latino gangs and delinquent bands selected the gang/band name itself as primary group. For “most important values in your life”, jihadists most frequently offered “Islam,” “Sharia,” or “Religion”; gang members most cited “honor” and “respect.” We opted for “Religion” as the primary value-set for jihadists and non-jihadists, and “Honor” for gangs and bands.

In consultation with prison psychologists, we selected six sacrifices considered extreme in the prison context, where all jihadists and the most violent gang offenders remain in solitary confinement: “To give up my prison income,” “To give up communications with my family,” “To move to another prison further away from my family,” “To stop taking part in activities that make me feel good,” “To lose commodities into prison,” and “To die for someone or for something.” Participants had to rate to what extent they consider each of these sacrifices as extreme on a scale ranging from 0 (low extreme) to 6 (high extreme). Participants rated the six sacrifices as highly extreme, with all means higher than the mid-point of the scale. We understand that willingness to die and kill is the overriding sacrifice that extremist rehabilitation programs hope to prevent. However, asking directly about these intentions can lead, and did lead (in our preliminary study), to difficulties in caring out the study (the participant could feel uneasy during the interview, he could prefer to abandon the interview, or he could even be aggressive with the interviewer). The analyses of the perception of extremism with regard to the other five sacrifices, as compared to willingness to fight and die, reliably allow us to use the scale as a proxy to assess the same motivation without incurring potentially insurmountable problems in executing the study.

Because some participants in the preliminary interviews, especially jihadists, expressed unease talking about “dying” and “killing,” we excluded willingness to die from the final survey with our full sample (Fig. S2). The remaining sacrifices concerned losing access to various material benefits or activities in prison and losing access to family.

Risk factors were rated: first with a three-item scale for perceived injustice, asking to what extent they perceive that Spain’s goal in condemning them to prison was vengeance, humiliation, and harm on the part of the Spanish state and prison authorities (*α* = 0.85): and second with a three-item scale for personal discrimination, asking to what extent the prisoner, in being a group member, has been personally a victim of discrimination, deprived of opportunities, and a victim of society (*α* = 0.80). Such sentiments relate to feelings of injustice, social exclusion, and the loss of personal significance^14^. Lastly, to probe protective factors, we measured identity fusion with the nation’s justice system (i.e., with Spain, security forces, and prison personnel, *α* = 0.71, see Supplementary) and family (i.e., father, mother, brothers, and sisters, *α* = 0.70).

## Analytical approach

Our analyses were performed in two stages. The first stage involved:

1.1. A qualitative analysis and classified the responses of the participants into thematic categories based on its relevance for previous research about radical intentions, rehabilitation programs and risk factors.
1.2. Registering expressions in favor and against each category.

For the second stage, we examined the following:

2.1. Correlations to explore the relation between the critical variables, and univariate analyses for testing the comparison of all the variables between the groups.
2.2. Repeated measure analyses on how each group of prisoners’ perceive their level of fusion with their primary reference group and value in the past and how they perceive their level of fusion in the present. We justify making repeated measure analyses for the perception of fusion in the past and present, either with the group or the value, instead of treating them as separated measures for several reasons: (a) we are not interested in the accuracy of the memory, but rather in the (subjective) perception of the cognitive representation of fusion at each time point; (b) the task does not focus on recall of episodic memories (i.e., remembering particular events and details), but rather what our participants believe, think they know, and conceive their identities to be; (c) the quasi-experimental design of our study, which compares participants from different groups, control for bias effect involved in past reflections, because such bias would affect all of the groups; and (d) the repeated measure analyses allows simultaneous testing of intergroup comparisons (between groups and for each time point), and intragroup comparisons (differences on the perceptions for each group between the past and the present), which enrich the empirical and theoretical contribution of the data).
2.3. Repeated measure analyses comparing the four groups in terms of their willingness to sacrifice for group and values.
2.4. A first mediation analyses testing the risk and protective factors relating the group with willingness to sacrifice for values in prison.
2.5. A second mediation analyses testing the risk and protective factors relating the group with perceived injustice in sentencing.

## Results

### Stage 1: Evidence and relative importance of selected analytic categories

The responses to the first stage of the interviews were randomly distributed among twenty-five researchers who were blind to the study’s theoretical framework, but who nevertheless were told to ask jihadists and non-jihadists about their relationship to “Muslims” and “Religion”, and to ask members of Latino gangs and delinquent bands about their relationship to their named group and to “Honor.” Nine investigators transcribed 514 h of recorded oral information, which two evaluators then independently classified into eight thematic categories: four categories based on Identity Fusion, and the Devoted Actor frameworks, as well as the recent meta-analyses identifying fusion^8^ and cherished values^2^ as top predictors of radical intentions (fusion with primary group, fusion with primary value, sacrifice for primary group, sacrifice for primary value); two based on a review of promising prison rehabilitation programs across the European Union^49^ (fusion with family, fusion with the national justice system); and two categories based on information that repeatedly appeared across interviews and have been also identified as strong risk factors^8^ (perceptions of injustice, perceptions of discrimination). The content analyses revealed 3031 sentences that corresponded to these categories. Each category was evaluated in terms of numerous thematic indicators (see Table S1 for category framework and thematic coding indicators). Evaluators registered judgments in favor and against inclusion in each category.

**Figure 1:**
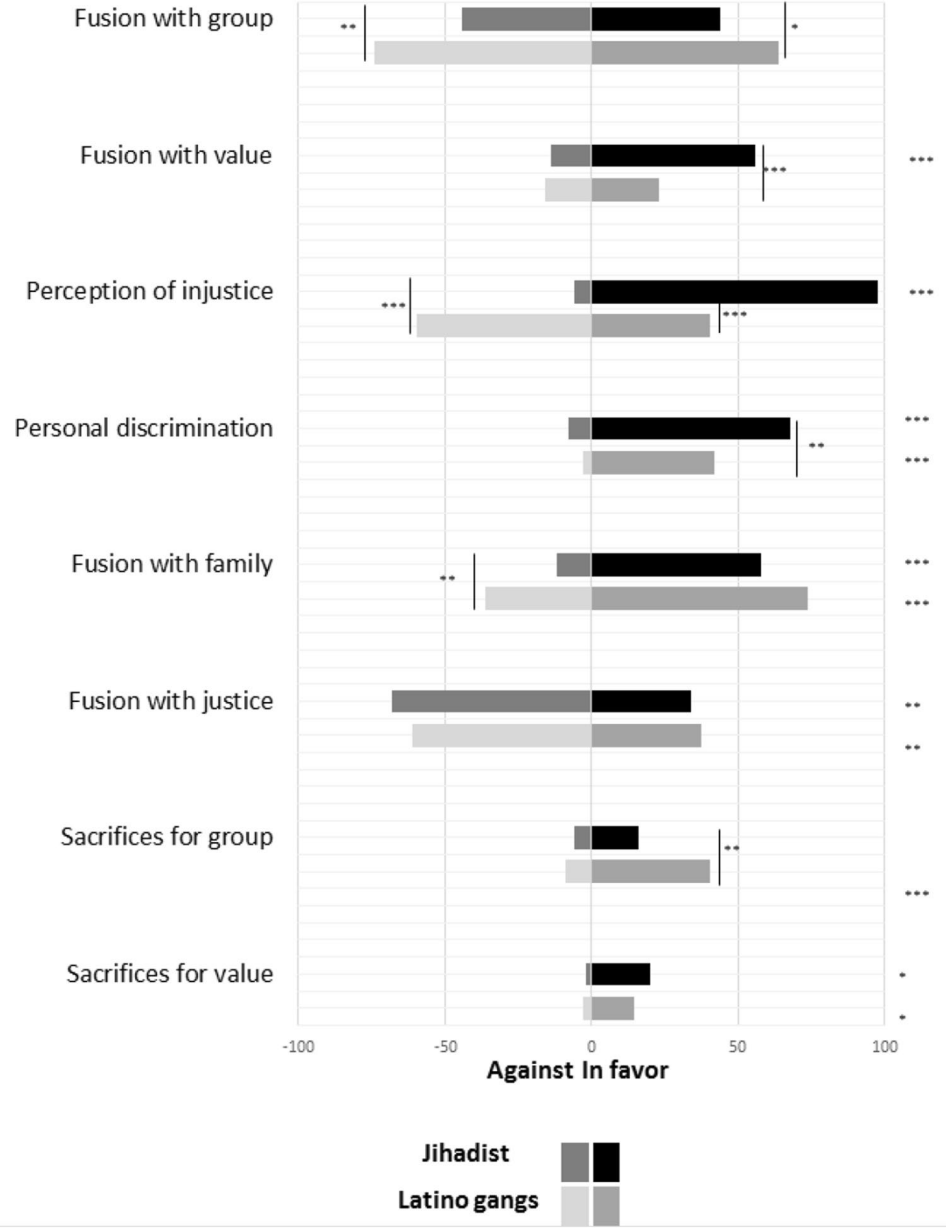
Percentage of jihadists versus Latino gang members providing at least one thematic confirmation for each of eight analytic categories. Right columns represent percentages of prisoners reporting judgements in favor of each category (e.g., high fusion with group); left columns represent percentages of prisoners reporting judgements against each category (e.g., low fusion with group). Asterisks inside the figure indicate significant differences between jihadists and gangs (in displaying more judgements of the category). Asterisks to the right of the figure indicate significant differences within each group (displaying more judgements in favor or against each analytic category). ***p < .001; **p < .01; * *p* < .05.

Tables S2–S9 provide evidence for the reality and relative importance of each set of paired categories (in favor versus against) for jihadists as compared to members of Latino gangs and others (non-jihadists, delinquent bands). Figure 1 illustrates the percentage of jihadists and members of Latino gangs who provided direct evidence for that category set (in terms of its thematic indicators). In contrast to gang members, jihadists show more evidence in favor of fusion with value as well as greater expression of injustice and personal discrimination (see also Fig. S4). Much the same pattern holds, even more pronounced, for jihadists versus the two other groups (Fig. S3, Fig. S5).

Especially in the jihadist interviews, there was an emerging narrative relating these sentiments: low fusion with the group (e.g., “I trust in God, but not in Muslims”), strong fusion with the value (e.g., “Islam is above my wife, my house, my mother, myself… I have chosen a way of life. Islam is a way of life”), perceptions of injustice (e.g., “I was accused of recruitment. But no one appeared at the trial to say ‘this person has recruited me’”), personal discrimination (e.g., “I am here because I am a Muslim, and those that committed the terrorist attack are Muslims. If I were someone else, I wouldn´t be in prison”), low fusion with the justice system (e.g., “What do the Spanish want? What does the justice system want? Do they want to sleep in peace or that no people in Spain are Muslim? In Spain they do not fight terrorism but rather Muslims”), strong fusion with the family (e.g., “I don´t trade my family for anything. Family is priceless”) and extreme sacrifices for the value (e.g., “I would die for my ideas. I would fight for what is my greatest happiness. I would abandon everything for my religion”).

### Stage 2: Responses to the questionnaire (for correlations, see Supplementary Tables S10–S13)

#### Fusion with group and value

**Figure 2:**
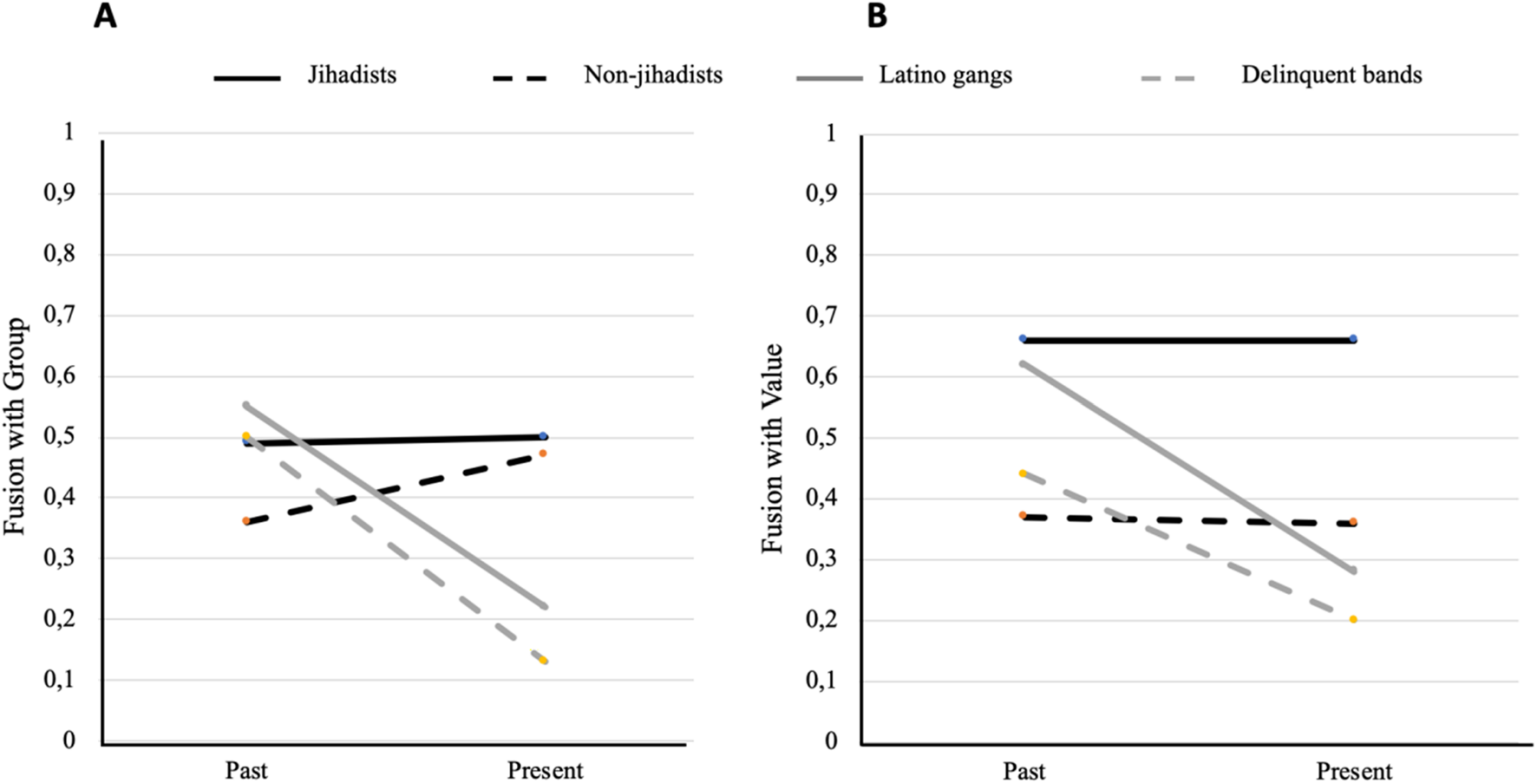
Prisoners’ perceived level of fusion with primary reference group (**A**) and value (**B**) over time (time of sentencing to present, average 56.28 months). Jihadists maintain, and non-jihadists increase, fusion with primary group (i.e., Muslims), whereas Latino gangs and delinquent bands decrease fusion with their primary criminal groups over time. Jihadist and non-jihadists maintain fusion with their primary value (i.e., religion), but jihadists show higher levels than non-jihadists, while gangs and bands show less fusion with value (i.e., honor) over time.

Figure 2 shows reported fusion levels for the four groups of prisoners, for group (A) and value (B). Repeated measure analyses on fusion with group in the past and in the present yielded a significant interaction (*F*(3,254) = 19.77, *p* < 0.001, *η*_*p*_^*2*^ = 0.19). Jihadists see their fusion with the group as constant, and non-jihadists increase fusion with the group, whereas Latino and non-Latino gang members report a sharp decrease (Fig. 2A). The same interaction was seen in fusion with value (*F*(3,242) = 10.83, *p* < 0.001, *η*_*p*_^*2*^ = 0.12). Although both jihadists and violent gang members were separated in prison, the former maintained their identity with the value (i.e., higher in the present than for the other groups), whereas the latter didn’t. Jihadists and non-jihadist Muslims were much more similar in their fusion with group than with values (Fig. 2B).

#### Willingness to sacrifice

**Figure 3:**
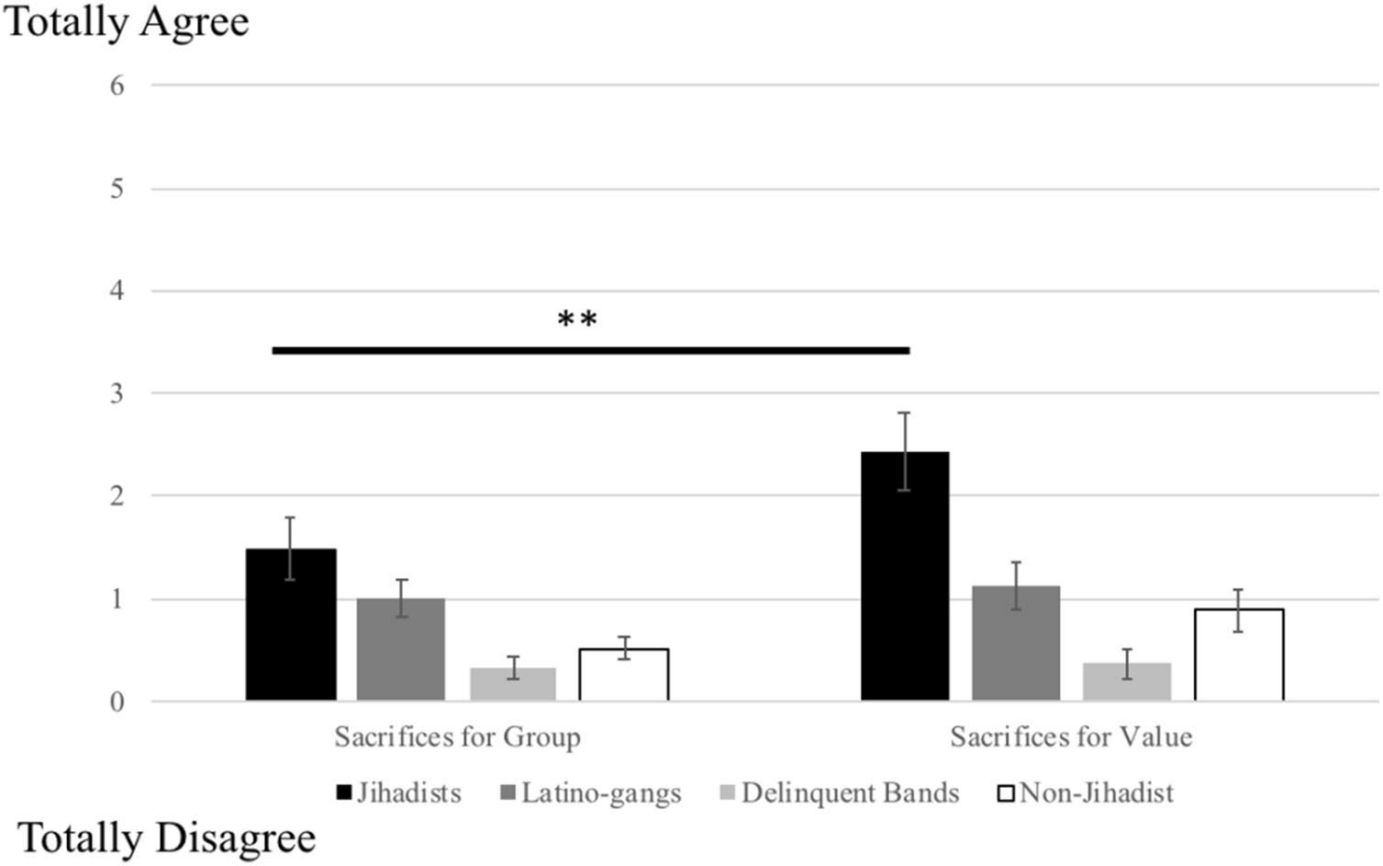
Sacrifice for group and for value. Jihadists are more willing to do costly sacrifices in prison for their more cherished group (Muslims) and value (Religion) than other criminals. And among jihadists, they are more willing to do costly sacrifices in prison for the value than for the group. **p < .01.

Repeated measure analyses for group and value (*α*s = 0.86 and 0.95) yielded a significant interaction (*F*(3,207) = 3.25, *p* = 0.023, *η*_*p*_^*2*^ = 0.05). Jihadists expressed greater willingness to sacrifice for their group than did gangs, non-jihadists and bands, and more sacrifices for the value that all three groups. In addition, only jihadists expressed more willingness to sacrifice for the value than for the group (*F*(3,207) = 12.94, *p* < 0.001, *η*_*p*_^*2*^ = 0.06) (Fig. 3).

A key factor in jihadists’ greater willingness to sacrifice for their group appears to be the superior importance that shared values have for jihadists in forging attachment to their group^59^. When participants were asked to what extent their shared values, shared positive experiences and shared negative (dysphoric) experiences contributed to their visceral commitment to the group (i.e., fusion), regression analyses for jihadists showed that shared values was the only factor significantly associated with group fusion both, perceived in the past and present (Fig. S6). The overriding importance of values, a characteristic of “devoted actors”^22,35,37^, is further evident among jihadists prisoners in that they expressed considerably greater willingness to sacrifice for their value than the other three groups did for theirs, and displayed greater willingness to sacrifice for their value than for their group.

To this point, results indicate that jihadists: (1) perceive a more stable feeling of fusion with their group and value over time than the other prisoners, (2) they sacrifice more for group and value than do others, (3) their sacrifices for value are greater than for group, and (4) shared value is a key enabling factor for their fusion with group.

Results concerning the remaining critical variables address the issue of why jihadists are more willing than other prisoners to make costly sacrifices, especially for value (i.e., risk factors) and what factors might reduce or mitigate their sacrifices (i.e., protective factors).

### Mediating roles of perceived injustice and personal discrimination

The qualitative analyses of the first stage showed that prisoners frequently referred to injustice and discrimination as experiences that might lead to the radicalization of individuals (i.e., risk factors). By contrast, they referred to the ties established with the family and to occasional examples of fair treatment by prison personnel, police and the courts as some of the most important factors in promoting the inmates’ reintegration (i.e., protective factors). These notable qualitative differences are consistent with the results of a recent metanalysis on cognitive and behavioral radicalization^8^. These differences mirror what we found in the interviews of the preliminary study, which further motivated us to quantitatively assess the possibility that perceived injustice and discrimination, and attitudes towards family and the justice system, could be reliable mediating factors in promoting or preventing radical behaviors associated with self-sacrifice.

Regarding the risk factors, the results of our qualitative analyses clearly showed that Islamist terrorist report the highest number of narratives indicating perception of injustice (see Fig. 1). Univariate analyses on perceived injustice in sentencing yielded a significant effect, (*F*(3,261) = 7.86, *p* < 0.001, *η*_*p*_^*2*^ = 0.08), indicating that jihadists perceive greater injustice in sentencing than do all other groups, and replicating the previous findings. Controlling for the time spent in prison, mediation analysis indicates that such perception of injustice in sentencing, in turn, seems to foster greater sacrifice for the jihadists’ value than is the case for gangs or other prisoner groups (see supplementary Figs. S7a, b).

Assuming that perceptions of injustice are often reliably associated with extreme behaviors, we wondered how the effects of injustice on willingness to sacrifice might be ameliorated. Regarding the protective factors, previous research indicates that efforts to undermine deeply-held values that people sacrifice for often backfire^23,35^. What, then, might explain a relation between perceived injustice that lessens willingness to sacrifice but leaves intact attachment to values? In addition to the recent prison review across the European Union^49^ that recommends prison staff training in recognition and respect for prisoners’ religious values, at least two previous lines of research intimate that a positive relationship with the justice system might ameliorate the effects of injustice on violence: (1) Low recidivism rates associated with prisoner de-radicalization programs in Iraq (after the Abu Ghraib scandal)^60^ and in Saudi Arabia^61^ suggest that direct engagement with governing authorities and prison staff who recognize and respect detainees’ religious values and are willing to discuss grievances are critical to program success; and (2) recent work on procedural justice indicates less criminal behavior, higher voluntary social compliance, and greater belief in the justice system’s fairness when authorities convey “respect, dignity, and concern” through interpersonal contact^62^. Accordingly, we considered that one factor in de-radicalization and disengagement could be fusion with the nation’s justice system. Using univariate analyses, we found that the jihadists and the non-jihadists in our study did indeed express lower levels of fusion than Latino-gangs and delinquent bands (*F*(3,265) = 6.98, *p* < 0.001, *η*_*p*_^*2*^ = 0.07).

**Figure 4:**
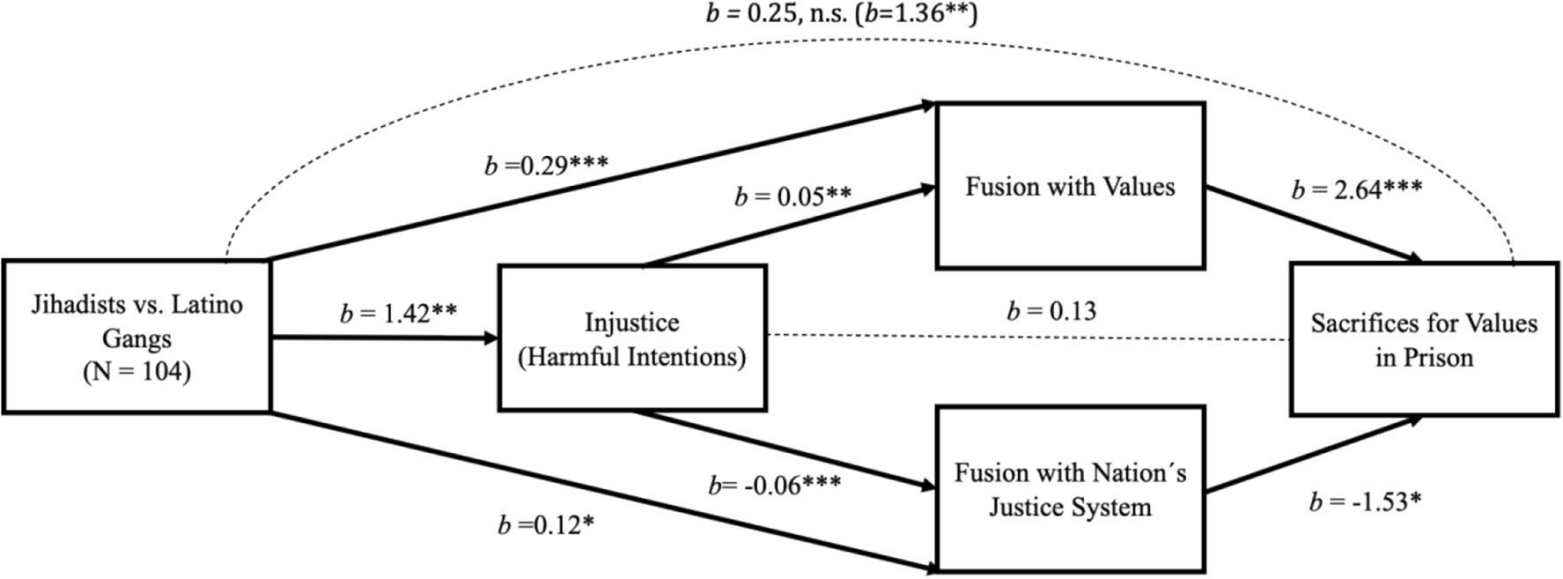
Mediation analysis showing: (1) jihadists express more feelings of perceived injustice in sentencing than do gang members; (2) their feelings of injustice increase willingness to sacrifice for values by increasing fusion with values; (3) lessening levels of perceived injustice increases fusion with the nation’s justice system, which in turn reduces willingness to sacrifice for (religious) values. *b* values are unstandardized β values; *p < .05; **p < .01; ***p < .001. Total effect: *b* = 1.36, 95% CI [0.53, 2.20]. Direct effect: *b* = 0.24, 95% CI [− 0.48, 0.99]. Indirect effects: Group > Injustice > Sacrifices: *b* = 0.19, 95% CI [− 0.06, 0.56]; Group > Fusion values > Sacrifices: *b* = 0.78, 95% CI [0.30, 1.38]; Group > Fusion justice system > Sacrifices: *b* = − 0.19, 95%CI [− 0.52, − 0.01]; Group > Injustice > Fusion values > Sacrifices: *b* = 0.20, 95%CI [0.05, 0.44]; Group > Injustice > Fusion justice system > Sacrifices: *b* = 0.13, 95% CI [0.02, 0.30].

When comparing jihadists versus Latino gangs, as well as the full sample of criminals (see supplementary Fig. S8), perceptions of injustice increase sacrifices for values in prison through two paths: (1) by increasing fusion with values, which is positively associated with sacrifices for values, and (2) by reducing fusion with the nation’s justice system, which is negatively associated with sacrifices for values. This mediation (Fig. 4), provides empirical evidence for three conclusions: (1) perceived injustice is related to sacrifices for values in prison through two different paths, fusion with values and fusion with Nation’s Justice System; (2) high levels of fusion with Nation´s Justice System are associated with low willingness to sacrifice; and (3) increasing fusion with Nation´s Justice System could plausibly reduce the effects of perceived injustice on willingness to sacrifice while leaving intact the attachment to values.

Cognizant of the strong relation between perception of injustice and sacrifices for values in prison, and that perception of injustice is a key factor differentiating Islamist terrorists and Latino-gangs, our next step was to determine what might affect the perceptions of injustice.

Regarding risk factors, results of our qualitative analyses also showed that Islamist terrorists reported higher levels of personal discrimination than did the other groups. (see Fig. 1). Univariate analyses on perceived personal discrimination yielded a significant effect, (*F*(3,237) = 12.85, *p* < 0.001, *η*_*p*_^*2*^ = 0.14); and, in line with the results of the qualitative analyses, jihadists perceive more personal discrimination than do all other groups.

Inasmuch as personal discrimination is also associated with extreme behaviors, we wondered how the effects of personal discrimination on willingness to sacrifice might be ameliorated. Previous work shows that family is the group most people are willing to die for^40^, and that devotion to family is one of the most cherished values in the Islamic world^26,54^. Univariate analyses on fusion with family yielded a significant effect, *F*(3,243) = 8.901, *p* < 0.001, *η*_*p*_^*2*^ = 0.10), indicating that Jihadists are more fused with family than are Latino gang members.

**Figure 5:**
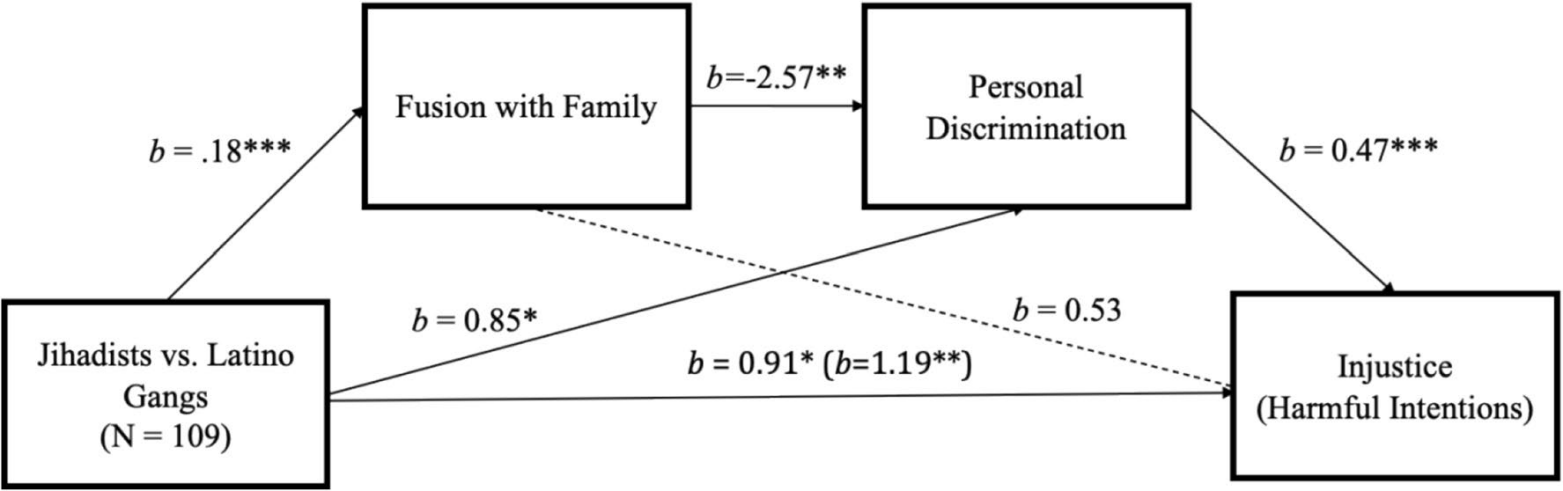
Mediation analyses showing: (1) jihadists express more fusion with family and personal discrimination than do gang members; (2) fusion with family reduces the perception of personal discrimination, which in turn is associated with less perceived injustice in sentencing. *b* values are unstandardized β values; *p < .05; **p < .01; ***p < .001. Total effect: *b* = 1.19, 95% CI [0.36, 2.02]. Direct effect: *b* = 0.91, 95% CI [0.07, 1.75]. Indirect effects: Group > Fusion family > Injustice: *b* = 0.09, 95% CI [− 0.28, 0.52]; Group > Discrimination > Injustice: *b* = 0.40, 95% CI [0.003, 0.83]; Group > Fusion family > Discrimination > Injustice: *b* = − 0.21, 95%CI [− 0.37, − 0.06].

When comparing jihadists versus Latino gangs, as well as the full sample of criminals (see supplementary Fig. S9), we found that jihadists perceived greater injustice because they also perceived greater personal discrimination; however, jihadists are more fused with the family, which is negatively related to personal discrimination. This implies that greater positive engagement with family (another key element of the prison rehabilitation programs in Iraq and in Saudi Arabia) leads to less susceptibility to feelings of personal discrimination and less perception of injustice, which is what the following mediation analysis reveals (Fig. 5).

## Discussion

Despite the policies of numerous nations that treat terrorism fundamentally as a criminal activity, there are compelling psychosocial differences between imprisoned Islamist terrorists and other prison inmates, including gang members with which Islamist terrorists are often compared. A theory-based understanding of these differences in terms of prisoners’ willingness to make costly sacrifices, validated by our direct observational and comparative study, has important *policy implications* for prisoner management (e.g., interventions programs for preventing radicalization and de-radicalization), but also significantly for societies-at-large: in particular, for lessening risks of recidivism, given the release of many hundreds of returning foreign ISIS fighters, and more generally for suggesting possibilities for lessening the impact and growth of terrorism.

Another substantive contribution of this study is that it is one of only a handful of studies that analyzes the factors underlying cognitive radicalization in a sample of terrorists under real-world conditions^26,63–65^ and, to our knowledge, the first such study of convicted terrorists in a prison environment. Although previous work and a recent meta-analysis on cognitive radicalization^8^ have suggested that the factors that we have tested in our study should work as risk or protective factors, such studies do not directly compare terrorists with other groups in a common environmental setting or jointly take into consideration risk and protective factors. In addition, our finding that mechanisms previously identified by researchers as factors affecting cognitive radicalization have been successfully tested in a real-world sample of criminal offenders should encourage future collaborations between those concerned by the academic as well as security and law enforcement communities.

Our main results, which are a product of mutually reinforcing qualitative and quantitative analyses, reveal that the nature of extremism for imprisoned jihadists, assessed in terms of willingness to self-sacrifice for primary group and value, is appreciably different from that of Latino gangs or other convicted criminals. The mediation models further indicate psychosocial risk factors that reliably favor greater willingness to sacrifice for values (i.e., perception of injustice and personal discrimination that boosts fusion with value), as well as protective factors that mitigate such sacrifice (i.e., fusion with family and the justice system). Together, these findings provide a theory-driven analysis of radicalization and de-radicalization factors that is empirically supported by direct, stepwise comparisons of rarely studied prison populations with outsized socio-political importance.

Our results conform with a recent review of prison programs across the European Union^49^, which surmises that the most promising “rehabilitation interventions” involve: (1) individualized psychological engagement with prison personnel to reduce prisoner sentiments of personal discrimination and social marginalization, (2) prison staff training in recognition and respect for prisoners’ religious values and in discussing alternative perspectives for preventing violence, and (3) actively engaging families and friends as partners in the disengagement process. Such interventions appear to be important factors in the EU’s relatively low jihadist recidivism rates^66^ (e.g., in Spain, 7% for a wide sample of jihadists^67^ vs. 45% for gang members^68^). Indeed, a principal reason Spain’s government supported our study was to find out if this surmise had scientific grounding, and what psychosocial variables might be involved, such as expressed commitments to primary values and groups, and willingness to sacrifice.

Our research indicates that policies for dealing with Islamist terrorists, inside prison and outside, should focus on what distinguishes them from other violent offenders and criminals: (1) greater commitment to their primary values, which is strongly associated with costly sacrifices; (2) longer-lasting commitment to their primary values and the group representing those values, undiminished by time in prison; and (3) stronger perception of unfair and hostile treatment by the state and mainstream society, which reinforces the link between commitment to value and group and willingness to sacrifice. These differences favor policies recognizing and respecting their values and value-driven allegiances to an extent mainstream society finds tolerable, but reorienting those values away from violence^69^. This is possible by reframing and reinterpreting values and offering alternative pathways for their realization (as when the meaning of religious canons and commandments is reconstrued for changing circumstances)^70^ and it is how some religious leaders have dissuaded suicide bombers without challenging their core beliefs and identity^26^. Our research also suggests that state authorities, prison staff, and families^71^ can help allay risk factors that foster radical behaviors associated with self-sacrifice (i.e., perceptions of injustice and discrimination) by favoring protective factors (contact with family, and training staff from the penitentiary system and law enforcement), so that abiding values can be reoriented to less extreme and belligerent behaviors that reduce terrorism’s appeal and growth.

A final caveat: It is possible that the age differences between prisoner groups, with jihadists being on average more than 8 years older than gang members, may render gang members less susceptible than jihadists to political awareness and militancy. Differences in age may also suggest pre-prison prevention, intervention, and suppression efforts attuned to different moments and activities in the life course^59^: for example, youth in a transitional stage of life, having left their genetic family to seek new associations of friends and fellow travelers, versus a more mature stage in life that may favor a return to traditional religion and family.

## Conclusion

Our research was intended to fill relevant gaps in the literature about violent radicalization and criminality by conducting an on-site study that directly compared different convict populations from the same prison environments: (1) inmates imprisoned because of jihadist terrorism, (2) a population of Muslims incarcerated because of reasons unrelated to terrorism, (3) a non-Muslim population of delinquent band members, and (4) members of Latino gangs. A principal aim was to better understand the similarities and differences between an important terrorist population and representatives of violent gangs with which terrorists have often been compared, although hitherto not directly under real-world field conditions. In doing so, we sought to illuminate the specific psychosocial factors that distinguish Islamist terrorists from other sorts of criminals by engaging a theoretical framework (Identity Fusion and the Devoted Actor) through a quasi-experimental design, testing it via a triangulation of qualitative and quantitative methods. The results provide empirical evidence for making inferences regarding the willingness to make costly sacrifices as a function of persistent fusion with the Islamist terrorists’ most cherished group and value (a persistence not found among the other criminal populations studied), as well as risk factors likely to increase such willingness (perception of injustice and personal discrimination that boosts fusion with value) and protective factors likely to decrease such willingness (fusion with family and the justice system).

On a policy level, these findings also have potentially important ramifications for the penitentiary system and society-at-large that could help to forestall recidivism, and to disengage from radicalization and recruitment into violent extremism more generally. The scope of the present study is restricted to Islamist terrorists, or jihadists, and as such cannot directly speak to other forms of subnational, national and transnational terrorism, such as the resurgence of extreme right wing and racial supremacist violence. Nevertheless, recent comparative analyses of ethno-nationalist and Islamist terrorism note important convergences with regard to key variables examined in the present study (devotion to group identity and ideological values, perceived injustice and discrimination), which likely render our findings relevant for wider comparative studies of terrorism and its relation to criminality^14,24–26^.A broader implication, and guiding principle, of our study was to show that a strong theoretical approach to pressing and tenacious real-world threats, such as a pervasive form of transnational terrorism, in ecologically valid field conditions, may help scientists and policymakers to come together in understanding how to mitigate such threats in the long term as well as in the here and now.

## Methods

Pre-registration of this study was not feasible for the following reasons: 1. After approaching several governments for permission to begin a comparative study of jihadists with Latino gang members and other prison populations, only the Spanish government allowed direct, unsupervised access to these populations under the condition that we commence the study immediately and proceed in a timely fashion (and with awareness that changes in the political regime or other developments could delay or halt the study). 2. Although we did have a theoretical framework to address the study, based on our previous behavioral and neuroimaging studies of extremist behaviors in different conflict areas and cultural contexts, only as the study progressed could we determine just what kinds of methods and measures might be acceptable to prison populations that had never been systematically compared before.

### Data source and ethical approval

This study was a primary data analysis based on in-depth face-to-face interviews and surveys in 35 Spanish prisons with 350 male convicts from 36 nationalities. Prospective participants were identified through the consultation of their corresponding prison files indicating the reason for their imprisonment and the group they belong. They were latter approached by a member of the staff (i.e., a psychologist or social educator) who privately asked them if they would like to take part in an investigation conducted by the Universidad Nacional de Educación a Distancia (Spain’s largest university) with the goal of exploring a series of questions about the belonging of inmates to different groups, their preferences about values important to them, and about their thoughts and feelings during their stay in prison. Those who agreed to participate were interviewed individually by one of the trained members of our research team in a private room within the prison complex, set up for the purpose of our research by prison staff to be free of outside recording systems and without any staff member in the room or in the corridors where the rooms were located. Participation was not rewarded.

When the interview started, and after repeating again the goals of the interviews, participants were asked to read and acknowledge understanding of a document on human subjects protection, and then provided a document of informed consent that they signed if agreeing to participate in the interview (see Supplementary materials, pp. 53–64). Participants were given details about (1) the nature, goals and procedure of the study; (2) the type of measures and questions included; and (3) the risks and benefits associated with the study, the voluntary nature of participation and the possibility to withdraw at any time without negative consequences. Participants also learned that participation would not have any positive or negative impact in their sentence or treatment in prison, that personal information shared with interviewers would be kept confidential, that all their responses would be anonymized and that, in addition to human subjects protections guaranteed by Spanish authorities, they would be protected under the protocols of the university’s ethical review board (see Supplementary, p. 52). Because measures and analysis also would involve researchers from Artis International, a research group dedicated to field-based scientific research on causes and solutions to intergroup violence, protocols were also subject to that institution’s human subjects review (Approval Number: 2019-0108; registered in Supplementary, p. 51). All the interviewers were trained to detect non-verbal signs of discomfort and lack of understanding and they inquired the participants about such signs and clarified any aspect of the study that had not been adequately grasped. All participants who exhibited signs of intellectual fragility, excessive anxiety and psychological disequilibrium or instability during the interview were automatically excluded from the study.

The interviews were audio-recorded by the interviewer with the permission of the inmates. None of the interviewees refused to be recorded. The recordings were transcribed without including any specific detail that might allow the personal identification of the participants and destroyed afterwards. We did not inquire for identificatory information in the survey. Participants were identified by a random code and all the data is kept exclusively in secure conditions by the UNED project director (Á. Gómez). The research abides to the terms of the Declaration of Helsinki. All methods were carried out in accordance with relevant guidelines and regulations. All experimental protocols were approved by a named institutional and/or licensing committee (ARTIS in U.S., and UNED in Spain). Informed consent was obtained from all subjects, and informed consent has been taken from the legal guardian/LAR.

The study was designed to compare four classes of prisoners readily distinguished by the Spanish Ministry of Interior’s filing system: *jihadists* (Islamist terrorists), *Latino gangs*, *non-jihadists* (Muslims unconnected with criminal organizations), and *delinquent bands* (local non-Muslim groups). Eleven participants were excluded owing to different mental illnesses, and twenty-one because they did not want to respond to most of the questions. In addition, at the end of the questionnaire participants were asked for a 3-item scale based on the MMPI questionnaire for liars detection^72^: “Sometimes I tell lies”, “Sometimes I think things too bad to talk about them”, and “Sometimes I tell bad things of other people”, on scales ranging from 0 (totally disagree), to 6 (totally agree), *α* = 0.72. Forty-one participants were excluded for the analyses because they responded “0” to the three items.

As a result, 277 participants were considered for the analyses:

- Jihadists (Islamist terrorists, *N* = 57, *M*_Age_ = 35.02, *SD* = 9.49, *M*_Confinement_ = 39.04 months, *SD* = 41.57), including returning ISIS foreign fighters and participants in the 2004 Madrid train bombings and 2017 vehicle attack on pedestrians in Barcelona and Cambrils;
- Latino Gangs (*N* = 79, *M*_Age_ = 35.77, *SD* = 9.51, *M*_Confinement_ = 55.14 months, *SD* = 51.82), including murderers and violent offenders from Ñetas (16.5%, Puerto Rican), Latin Kings (15.2%, largest Hispanic street gang worldwide), Trinitarios (15.2%) and DDP (8.9%, Dominican gangs founded in New York), and MS-13 (2.5% Mara Salvatrucha, founded in Los Angeles);
- Non-jihadists Muslims unconnected with criminal organizations, (*N* = 94, *M*_Age_ = 35.77, *SD* = 9.51, *M*_Confinement_ = 55.14 months, *SD* = 51.82); and
- Delinquent bands (local non-Muslim groups, *N* = 47, *M*_Age_ = 47.85, *SD* = 10.04, *M*_Confinement_ = 82.23 months, *SD* = 69.05).

The latter two groups mainly involved petty criminal activity (drug trafficking, robbery, assault and battery, etc.).

The first stage of the study consisted of an interview with each participant and subsequent content analysis. Interviews were randomly distributed among twenty-five researchers who were blind to the study’s theoretical framework or policy goals. Interviewers were instructed to impose minimal structure but to elicit participants’ daily routines and life stories, as well as the most important groups and values participants identified with. Thirty-seven interviews were excluded from content analysis in the first stage (but not necessarily from survey analysis in the second stage) because of poor audio recording or lack of final authorization to make content available for analysis. Each interview lasted approximately 1,5–2 h.

In the initial coding, researchers listened to each interview and categorized the narrative using pre-established categories based on the theoretical frameworks of Identity Fusion and the Devoted Actor (i.e., fusion with group and value, sacrifice for group and value), promising factors in EU-wide reports of prisoner rehabilitation (support from family, and from prison and law enforcement personnel), and recurrent complaints of injustice and personal discrimination. Researchers coded the interviews for specific concepts (thematic indicators) falling under these categories, counting the number of times a specific concept appeared under a given category in accordance with coding rules to distinguish between word segments. All category-related word segments and sentences were hand-coded (communication issues precluded use of computer coding, especially when Spanish was not the native language). A total of 3031 sentences/segments were obtained (jihadist, *N* = 774; non-jihadist Muslim, *N* = 779; Latino gang, *N* = 1118; delinquent band, N = 360). Some segments included information that fit into more than one category.

In the next coding cycle, two independent judges each read the sentences/segments and categorized them into the eight pre-established category sets (fusion/no fusion with group, fusion/no fusion with value, etc.). The judges did not consider the category assigned by the initial coder. In the final coding cycle, judges 1 and 2 discussed any disagreement, and turned to the initial codes to help resolve it. Unresolved disagreements were classed “other.” Across categories, inter-judge reliability proved adequate (Cohen’s *κ* = 0.96).

The study’s second stage involved preset survey questions and measures. Participants were introduced to an iPad, and we used our own survey platform for offline data collection and dynamic measures^22,73^. Before starting the questionnaire, participants unfamiliar with tablets were introduced to the iPad’s functioning. Participants were free to respond to any of the questions, or leave blank those that made them uncomfortable, which accounts for missing cases regarding some of the variables. They could end the meeting at any time for any reason.

### Overview of measures in the second stage of the study

#### Identity fusion

Following previous research, we asked participants to indicate their level of fusion with the appropriate primary reference group and value^38^, both for the time of the interview (present) and how they perceived that it was their level of fusion at the time of their sentencing (past). We used fusion with values, such as honor and religion, instead of our usual measures of sacred values (immunity to material tradeoffs) because Islamist terrorist prisoners became insulted and angry by offers to compromise their religious values for material benefits, and because previous studies showed that fusion with values is a strong predictor of sacred values and vice versa^41^. Participants used the Dynamic Identity Fusion Index (DIFI)^73^, which shows a figure formed by two circles of different sizes on a screen. The small circle represents ‘‘the self’’ (or “me”), and the big circle represents ‘‘the group’’ or “a value.” The respondent places a finger on the small circle and moves it towards the big circle. The degree of overlap between the circles goes from 0 (not fused at all = circles remain separate) to 1 (fully fused = small circle moved entirely into big circle).

In measuring *fusion with family*, participants were asked independently for their degree of fusion with their father, mother, brothers, and sisters, (*α* = 0.70). In measuring *fusion with nation´s justice system*, participants were asked to rate: (1) their fusion with Spain, (2) fusion with the forces and bodies of the State Security police, civil guard, and (3) fusion with the prison staff (*α* = 0.71).

*Costly sacrifices for group and for value* were measured with the 5-item series from the preliminary study, (*α*s = 0.87 and 0.95 for sacrifices for group and value, respectively).

*Causes of fusion with the group* were assessed by asking participants on a scale from 0 (totally disagree) to 6 (totally agree), to what extent they started to feel attached to their most cherished group on 3 items: "Because we shared important values and ideas,” “Because we shared intense positive experiences,” and “Because we shared intense negative experiences.”

*Perceived injustice in sentencing* was measured by a 3-item series asking to what extent they perceive that Spain’s goal in condemning them to prison was vengeance, humiliation, and harm on the part of the Spanish state and prison authorities on scales ranging from 0 (totally disagree) to 6 (totally agree), (*α* = 0.85).

*Perceived Personal Discrimination* was measured by a 3-item series where participants were asked on scales ranging from 0 (totally disagree) to 6 (totally agree), to respond to the following items: “I have personally been a victim of discrimination for being (group member),” “I consider myself a person who has been deprived of opportunities for being (group member),” and “I feel like I am a victim of society for being (group member),” where group member refers to Muslin, Gang, or Band (*α* = 0.80)^74^.

### Analyses of individual-level data

Correlational analyses were used to examine the relation between critical variables for the full sample as well as independently for each of the four prisoner groups. Univariate analyses were conducted to examine the differences on each variable for each of the four groups. Univariate instead of multivariate analyses were conducted because not all participants responded to all the questions, thus reducing the sample from a full comparison; nevertheless, alternative multivariate analyses yielded the same conclusions.

Repeated measure analyses with group as inter-subject factor, and (perceived) past and present as within factor, were conducted for fusion with group and fusion with value (controlling for the time spent in prison). A similar repeated measure analyses with group as inter-subject factor, and costly sacrifices for group and value as within factor, was conducted for costly sacrifices for group and value (controlling for the time spent in prison).

We applied a linear regression analyses to test the role of shared values, shared positive experiences and shared negative (dysphoric) experiences on participants’ visceral commitment to the group (i.e., fusion).

A series of linear mediational analyses was used to assess the roles of perceived injustice in sentencing, and perceived personal discrimination, in relation to fusion with groups, fusion with values, and willingness to sacrifice. In each case, we employed a bootstrapping test (5000 boots) with PROCESS^75^, comparing jihadists with Latino gang members as well as jihadists with the full sample, and controlling for time spent in prison. The first bootstrapping test (Model 4) was used to evaluate whether the differences between groups in terms of their sacrifices for values in prison was mediated by injustice (harmful intentions). The second test (Model 81) was used to investigate whether the differences between groups in terms of their willingness in prison to sacrifice for their value was mediated first by injustice (harmful intentions), and second by the effect of injustice on fusion with values and fusion with the national justice system. Finally, a third bootstrapping test (Model 6) was used to explore whether the differences between the groups regarding prisoners’ perceptions of injustice was mediated first by fusion with family, and second by perceptions of personal discrimination.

## Supplementary Information

Supplementary Information.
