# Supplementary material for: Willingness to sacrifice among convicted Islamist terrorists versus violent gang members and other criminals
Source: Sci Rep. 2022 Feb 16;12:2596. doi: 10.1038/s41598-022-06590-0 (PMC8850476; doi:10.1038/s41598-022-06590-0)
Supplement: Supplementary file 1 — Supplementary Information. [file 41598_2022_6590_MOESM1_ESM.docx]

Supplementary Materials for

**Willingness to Sacrifice Among Convicted Islamist Terrorists versus Violent Gang Members and Other Criminals**

**Angel Gómez^1,2,†^, Scott Atran^1,3,4,†,^*, Juana Chinchilla^2^, Alexandra Vázquez^1,2^, Lucia López-Rodríguez^1,5^, Borja Paredes^2,6^, Mercedes Martínez^2^, Laura Blanco^2^, Beatriz Alba^2^, Hend Bautista^2^, Saulo Fernández^2^, Florencia Pozuelo-Rubio^7^, José Luis González-Álvarez^8^, Sandra Chiclana^7^, Héctor Valladares-Narganes^7^, María Alonso^7^, Alfredo Ruíz-Alvarado^7^, José Luis López-Novo^7^, Richard Davis^1,3,9^**

Correspondence to: agomez@psi.uned,es

**This file includes:**

Preliminary Study

Main Study

Supplementary Figs.S1 to S9b

Supplementary Tables S1 to S21

**Others:**

Human Subjects Protection Documents

Preliminary Study

Methods

We first conducted a preliminary study to determine the primary groups and value-sets to be used in the survey, and a scale of costly sacrifices in prison.

Participants. Fifty-two male inmates (*M*_Age_ = 25.52, *SD* = 7.71) from four prisons in Spain participated in the preliminary study (3 jihadists, 8 Muslims classed by authorities as “under risk of radicalization,” 1 non-jihadist Muslim, 36 Latino gang members, 5 petty criminal delinquents). Participants were from 8 nationalities (Spain 22, Dominican Republic 10, Morocco 9, Ecuador 6, Bolivia 2, Belarus 1, Panama 1 Argentina 1). Their time spent in prison averaged some 30 months (*M*_Confinement_ = 29.64, *SD* = 36.29).

Procedure. Prospective participants were asked if they would like to take part in an investigation conducted by the Universidad Nacional de Educación a Distancia (Spain’s largest university) with the goal of testing a series of preliminary questions and surveys for a subsequent study about the belonging of inmates to different groups, their preferences about several values, and about their thoughts and feelings during their stay in prison. Participants were interviewed individually by one of the trained members of our research team in a private room within the prison complex, set up for the purpose of our research by prison staff to be free of video or audio recording systems and without any staff member in the room or in the corridors where the rooms were located. When the interview started, and after repeating again the goals of the interviews, participants were asked to read and acknowledge understanding of a document on human subjects protection, and then provided a document of informed consent that they signed if agreeing to participate in the interview. Participants learned that all their responses would be anonymous and that, in addition to human subjects protections guaranteed by Spanish authorities, they would be protected under the protocols of the university’s ethical review board. Participants were then asked: “which are the most important groups in your life, and why?”; and “which are most important values in your life, and why?” Finally, participants were asked to respond to a scaled series of potential sacrifices in prison by estimating how costly each sacrifice would be for them.

Results

*Primary groups and value-sets.* Participants were asked about the most important group and the most important value in their life, and the reasons why they choose such group and value. Participants were told to not consider “family” as group or value because previous research indicates that the family is the group that most people are willing to die for, and individuals usually consider family as a group but also as a value ^[1,2]^. Jihadists, Muslims under risk of radicalization, and the non-jihadist Muslim participants identified “Muslims” and “Religion” (also “Islam” and “Sharia”) as the most important group and value for them. Latino gang members and delinquents identified the name of their group, and “honor” or “respect” as the most important group and value for them.

Examples illustrating that for jihadists “Muslims” is the most important group are as follows: “All the Muslims in the world are my family. When I see an old woman, she is my mother; when I see an older woman, she is my grandmother… when I see a girl, she is my daughter"; “I would kill for the Muslims no matter how they are”; “I would die for my family. I would also protect my family ... and the family is ... all Muslims, because we are brothers, you have to defend them too”; “My goal in my life is dying Muslim!”

Examples illustrating that for jihadists the most important value is “Religion”: “I would go back to prison because defending religion is my way of life. I don’t care what others think, losing my life, my family...”; "Religion must be taken very seriously. I have watched imams on TV who talk about religion as if they were talking about a way of life. It is not just a way of life; Islam is not just a way of life. […] There are people who cannot interpret religion well. […] It is more than a way of life.”

Examples illustrating that for Latino gangs and delinquent bands the most important group is “the gang”: "You really live for them" (referring to the band and its members); "We are family... we are family... I have cried, I have cried. I've been to everyone's funerals. And the day I leave from here (prison), I will go to the cemetery to see them"; "All those in the group are brothers"; "I gave my blood and my freedom" (for them, in reference to the band and its members); “For me it means a lot the Latin Kings. It means a lot. […] I have belonged to them (to the Latin Kings) all my life and I carry it in my heart. […] I have the boys in my heart”; "When I get out (from prison), for some it is sure that they are going to die… What you need to know is how to die. It is the truth; you have to know how you want to die… as a Latin King… (pause) but never before your children."

Examples illustrating that for Latino gangs and delinquent bands the most important value is “honor”: "Within the group, honor is all, do you understand? It is like a rule, a main point. […] Honor is inside the group”; “People's code of honor is easy because when… (pause) you swear,… do you understand me? You are inside the other people (the members of the gang), do you understand me? As your brother I promise you that I will be with you in good times and bad, and if you are not there tomorrow and you are missed, I will be with your family. That is what happens.”

*Costly sacrifices in Prison*. Next, participants were introduced to a list of six items representing different examples of costly sacrifices for inmates that were selected in advance by four prison psychologists, each with more than a decade of experience: “To give up my prison income,” “To give up communications with my family,” “To move to another prison further away from my family,” “To stop taking part in activities that make me feel good,” “To lose commodities into prison,” and “To die for someone or for something.” Participants had to rate to what extent they consider each of these sacrifices as extreme on a scale ranging from 0 (low extreme) to 6 (high extreme). Participants rated the six sacrifices as highly extreme, with all means higher than the mid-point of the scale, *Means* from 3.94 to 5.03, all *p*s < .035. Participants rated “To give up communications with my family,” as more extreme than “To die for someone or for something,” *M* = 5.31, *SD* = 1.46 vs. *M* = 4.54, *SD* = 2.24, *t*(51) = 2.61, *p* = .012 (see Fig. 1).

Main Study Supplementary Details

Methods

We followed the same general protocols as in the preliminary study while conducting face-to-face interviews in 35 Spanish prisons (Fig. S2 includes the distribution of participants across prisons), except that interviews were audio-recorded with permission of the inmates. The main study had two stages, combining qualitative and quantitative analyses: minimally-structured interviews followed by well-structured surveys and measures.

**Participants.** Three hundred and fifteen male inmates participated in the present study. Eleven participants were excluded owing to different mental illnesses; and twenty-one because they did not want to respond to most of the questions. In addition, at the end of the questionnaire participants answered to a 3-item scale adapted from the MMPI questionnaire for liars detection by Hathaway and McKinley (1951): “Sometimes I tell lies”, “Sometimes I think things too bad to talk about them”, and “Sometimes I tell bad things of other people”, on scales ranging from 0 (totally disagree), to 6 (totally agree), *α* = .72. Forty-one participants were excluded for the analyses because they responded “0” to the three items. Then, 277 participants were considered for the analyses: 57 jihadists (*M*_Age_ = 35.02, *SD* = 9.49; *M*_Confinement_ = 39.04 months, *SD* = 41.57), 79 Latino gangs (*M*_Age_ = 26.75, *SD* = 6.40; *M*_Confinement_ = 47.37 months, *SD* = 48.02 ), 94 non-jihadists Muslims (*M*_Age_ = 35.77, *SD* = 9.51; *M*_Confinement_ = 55.14 months, *SD* = 51.82), and 47 Delinquent bands (*M*_Age_ = 47.85, *SD* = 10.04; *M*_Confinement_ = 82.23 months, *SD* = 69.05). In the case of Latino gangs, participants belonged to different groups, among others, 16.5% Ñetas, 15.2% Latin King, 15.2% Trinitarios, 8.9% DDP (Dominicans Don´t Play), and 2.5% M-13.

**Stage 1. Procedure.**

In the interviews, participants were asked to describe a normal day in their prison life, from awakening until day’s end. Next, they were asked to relate their life history from childhood to the present. Interviews were randomly distributed among nine researchers who were blind to the study’s theoretical framework, but who nevertheless were told to ask Jihadists and non-Jihadists about their relationship to “Muslims” and “Religion,” and to ask members of Latino gangs and Delinquent bands about their relationship to their named group and to “Honor”.

From the 277 male convicts interviewed, 37 participants were excluded from the qualitative analysis due to different motives (e.g., participants did not give their authorization, poor-quality recording), yielding a total of 240 interviews (jihadist, *N* = 50; Latino gang, *N* = 69; non-jihadist Muslim, *N* = 80; and delinquent band, *N* = 41).

Two evaluators independently classified 514 hours of recorded oral information into eight sets of paired categories: four sets based on the theoretical approaches of Identity Fusion and the Devoted Actor: (fusion/no fusion with primary group, fusion/no fusion with primary value, sacrifice/no sacrifice for primary group, sacrifice/no sacrifice for primary value); two sets based on a review of promising prison rehabilitation programs across the European Union^[3]^ (fusion/no fusion with family, fusion/no fusion with the national justice system); and two sets based on information that repeatedly appeared across interviews (perceptions of injustice/justice, perceptions of discrimination/no discrimination). Each category was evaluated in terms of numerous thematic indicators (Table S1). Each interview lasted approximately 2 hours.

All category-related word segments and sentences were hand-coded (communication issues precluded use of computer coding, especially when Spanish was not the native language). A total of 3,031 sentences/segments were obtained (jihadist A, *N* = 774; non-jihadist Muslim, *N* = 779; Latino gang, *N* = 1118; delinquent band, *N* = 360), (see Tables S2-S9 for results, and Tables S10 to S18 for examples of thematic indicators).

**Stage 1 Results.**

Table S2 shows the number and percentage of participants of each group that cited at least one thematic indicator related to each category. We conducted a series of McNemar tests among jihadists, Latino gangs, and others (non-jihadists and delinquent bands) and within each pair of categories (e.g., thematic indicators for fusion vs. thematic indicator for non-fusion). The goal was to test whether thematic indicators related to one pole (e.g., fusion) were cited by more participants than thematic indicators related to the other pole (e.g., non-fusion).

As Table S3 shows, among jihadists, thematic indicators of fusion (vs. non-fusion) with the value, injustice (vs. justice), personal discrimination (vs. non-personal discrimination), fusion (vs. non-fusion) with family, non-fusion (vs. fusion) with the Nation’s Justice System, and sacrifice (vs. non-sacrifice) for the value were cited by a higher percentage of participants.

Among Latino gangs, thematic indicators of personal discrimination (vs. non-personal discrimination), fusion (vs. non-fusion) with family, non-fusion (vs. fusion) with Nation’s Justice System, and sacrifice (vs. non-sacrifice) for the group and the value, were cited by a higher percentage of participants.

Among Latino gangs, non-jihadist, and delinquent bands altogether, thematic indicators of non-fusion (vs. fusion) with the group, personal discrimination (vs. non-personal discrimination), fusion (vs. non-fusion) with family, non-fusion (vs. fusion) with the Nation’s Justice System, and sacrifice (vs. non-sacrifice) for the group were cited by a higher percentage of participants.

**Comparisons between jihadists and Latino gangs who cited at least one thematic indicator related to each category.**

To check whether there were differences between jihadists and Latino gangs regarding the (non)appearance of thematic indicators related to each category we conducted a series of logistic regressions. The predictor was the group (0 jihadist, 1 gangs) with gangs as the reference group. As Table S4 shows, there were several significant differences between jihadists and Latino gangs. As compared to Latino gangs, jihadists were less likely to report at least one thematic indicator related to fusion and non-fusion with the group, justice, non-fusion with family and willingness to sacrifice for the group. In contrast, jihadists were more likely than Latino gangs to report at least one thematic indicator related to fusion with the value, injustice, and personal discrimination.

**Comparisons between jihadists and others (Latino gangs, non-jihadists, and delinquent bands) who cited at least one thematic indicator related to each category.**

To check whether there were differences between jihadists and the other three groups regarding the (non)appearance of thematic indicators related to each category, we conducted a series of logistic regressions. The predictor was the group (0 jihadist, 1 other) with others as the reference group. As Table S5 shows, jihadists were less likely than others to report at least one thematic indicator related to non-fusion with the group and to justice. In contrast, jihadists were more likely than others to report at least one thematic indicator related to fusion with the value, injustice, personal discrimination, non-fusion with the Nation’s Justice System, and willingness to sacrifice for the value. Table S6 shows the number of thematic indicators related to each category for each group as well as the mean sand standard deviations.

We conducted a series of Wilcoxon signed-rank tests within each group (jihadists, Latino gangs, and others) and within each pair of categories (e.g., thematic indicators for fusion vs. thematic indicator for non-fusion) to compare the number of thematic indicators. The goal was to test whether participants cited more thematic indicators related to one pole (e.g., fusion) than to the other pole (non-fusion). As Table S7 shows, jihadists cited more thematic indicators of fusion (vs. non-fusion) with the value, injustice (vs. justice), personal discrimination (vs. non-personal discrimination), fusion (vs. non-fusion) with family, non-fusion (vs. fusion) with the Nation’s Justice System, and sacrifice (vs. non-sacrifice) for the group and for the value. Latino gangs cited more thematic indicators of personal discrimination (vs. non-personal discrimination), fusion (vs. non-fusion) with family, non-fusion (vs. fusion) with the Nation’s Justice System, and sacrifice (vs. non-sacrifice) for the group and for the value. Finally, Latino gangs, non-jihadists and delinquent bands together cited more thematic indicators of non-fusion (vs. fusion) with the group, personal discrimination (vs. non- discrimination), fusion (vs. non-fusion) with family, non-fusion (vs. fusion) with the Nation’s Justice System, and sacrifice (vs. non-sacrifice) for the group.

**Comparisons between jihadists and Latino gangs regarding the number of thematic indicators related to each category.**

To check whether there were differences between jihadists and Latino gangs regarding the number of thematic indicators related to each category we conducted a series of Poisson regressions. The predictor was the group (0 jihadist, 1 gangs) with gangs as the reference group. As Table S8 shows, there were several significant differences between jihadists and Latino gangs. As compared to Latino gangs, jihadists tended to report less thematic indicators related to fusion and non-fusion with the group, justice, fusion and non-fusion with family and sacrifice for the group. In contrast, jihadists tended to report more thematic indicators related to fusion with the value, injustice, personal discrimination and no personal discrimination, and sacrifice for the value.

**Comparisons between jihadists and other groups regarding the number of thematic indicators related to each category.**

To check whether there were differences between jihadists and the other three groups regarding the number of thematic indicators related to each category we conducted a series of Poisson regressions. The predictor was the group (0 jihadist, 1 others) with others as the reference group. As Table S9 shows, there were several significant differences between jihadists and the other three groups. As compared to others, jihadists tended to report less thematic indicators related to non-fusion with the group and justice. In contrast, jihadists tended to report more thematic indicators related to fusion with the value, injustice, personal discrimination, non-fusion with the Nation´s Justice System and sacrifice for the value.

Stage 2 Procedure.

Participants responded to a questionnaire including the following measures: *Fusion with group and with value, Costly sacrifices for group and for value*, *Perceived injustice in sentencing*, *Perceived personal discrimination*. Tables S18-S20 include the correlation between all variables independently for each of the four groups of participants, and Table S21 includes Univariate Analyses for each of the outcome measures on each group

**Stage 2 Results**.

*Fusion with group***.** Repeated measures analyses on prisoners’ fusion with group in the past (at time of sentencing) and in the present (at time of interview), yielded a significant interaction (*F*(3,254) = 19.769, *p* < .001, *η_p_^2^* = .19), showing that the perception of fusion with group for jihadists is constant over time, *M*_Past_ = 0.49, *SD* = 0.46 vs. *M*_Present_ = 0.50, *SD* = 0.44, *p* = .92, increases somewhat for non-jihadists, *M*_Past_ = 0.36, *SD* = 0.45 vs. *M*_Present_ = 0.47, *SD* = 0.46, *p* = .023, and decreases markedly for gangs *M*_Past_ = 0.55, *SD* = 0.46 vs. *M*_Present_ = 0.22, *SD* = 0.37, *p* < .001, and bands *M*_Past_ = 0.49, *SD* = 0.46 vs. *M*_Present_ = 0.514 *SD* = 0.31, *p* < .001. There was also a significant main effect of the repeated measure, *F*(1,254) = 7.835, *p* <. 001, *η_p_^2^* = .03. No other main or interaction effects were significant, *p*s > 0.13

*Fusion with value*. Repeated measures analyses on fusion with value in the past and in the present also yielded a significant interaction (*F*(3,242) = 10.827, *p* < .001, *η_p_^2^* = .12), showing that the perception of fusion with value is constant over time for jihadists *M*_Past_ = 0.67, *SD* = 0.41 vs. *M*_Present_ = 0.67, *SD* = 0.39, *p* = .98, and non-jihadists *M*_Past_ = 0.36, *SD* = 0.46 vs. *M*_Present_ = 0.36, *SD* = 0.44, *p* = .84, and strongly decreases for gangs *M*_Past_ = 0.62, *SD* = 0.43 vs. *M*_Present_ = 0.28, *SD* = 0.42, *p* < .001, and bands *M*_Past_ = 0.42, *SD* = 0.47 vs. *M*_Present_ = 0.19, *SD* = 0.36, *p* < .001. There was also a significant main effect of group, *F*(3,242) = 9.128, *p* < .001, *η_p_^2^* = .10, and a main effect of the repeated measure, *F*(1,242) = 10.931, *p* <.001, *η_p_^2^* = .04. No other effects were significant, *p*s > 0.10.

*Fusion with family***.** Univariate analyses on fusion with family yielded a significant effect, *F*(3,243) = 8.901, *p* < .001, *η_p_^2^* = .10). Jihadists are more fused with family than Latino gangs, *M* = 0.96, *SD* = 0.11, vs. *M* = 0.78, *SD* = 0.22, *p* <.001, but not than bands *M* = 0.89, *SD* = 0.20, *p* = .713, or non-jihadists *M* = 0.88, *SD* = 0.20, *p* = .226. Latino-gangs were less fused with family than bands, *p* = .057, and non-jihadists, *p* = .006.

*Fusion with Nation´s Justice System***.** Univariate analyses on fusion with Nation´s Justice System yielded a significant effect, *F*(3,265) = 6.977, *p* < .001, *η_p_^2^* = .07). Non-jihadists displayed the highest level of fusion with Nation´s Justice System, *M* = 0.59, *SD* = 0.33, compared to Latino gangs *M* = 0.38, *SD* = 0.26, *p* <.001, and bands *M* = 0.42, *SD* = 0.34, *p* = .001, but they did not differ from jihadists, *M* = 0.46, *SD* = 0.34, *p* < .10.

*Willingness for Costly Sacrifices for group and value.* Repeated measures analyses on willingness to sacrifice for group and value yielded a significant interaction (*F*(3,207) = 3.245, *p* = .023, *η_p_^2^* = .05). Jihadists expressed greater willingness to sacrifice for their group than did gangs, *M* = 1.49, *SD* = 1.90 vs. *M* = 0.97, *SD* = 1.50, *p* = .05, non-jihadists *M* = 0.52, *SD* = 0.87, *p* < .001, and bands, *M* = 0.33, *SD* = 0.72, *p* < .001. Gangs also expressed more willingness to sacrifice for the group than did non-jihadists, *p* = .047, and bands, *p* =.012. Jihadists expressed more sacrifices for values than did gangs, *M* = 2.44, *SD* = 2.47 vs. *M* = 1.09, *SD* = 1.82, non-jihadists *M* = 0.89, *SD* = 1.65, and bands, *M* = 0.37, *SD* = 0.94, all *ps* < .001; and gangs expressed more sacrifices for values than bands, *p* = .043. The main effects of group and the repeated measure were significant, *F*(3,207) = 10.542, *p* < .001, *η_p_^2^* = .13, and *F*(1,207)=7.037, *p* = .009, *η_p_^2^* = .03, respectively.

*Factors of fusion with the group.* Repeated measures analyses were performed on three potential factors of fusion: shared values, shared positive experiences, and shared negative experiences. The analyses yielded a significant interaction (*F*(3,223) = 13.46, *p* < .001, *η_p_^2^* = .15). For jihadists, shared values were the main factor of fusion with the group, *M* = 4.16, *SD* = 2.10, significantly higher than shared positive experiences, *M* = 2.60, *SD* = 2.42, and negative experiences, *M* = 2.11, *SD* = 2.49, *p*s < .001. The difference between positive and negative experiences was not significant, *p* = .19. For non-jihadists, shared values were the main factor of fusion with the group, *M* = 2.70, *SD* = 2.31, significantly higher than shared positive experiences, *M* = 1.88, *SD* = 2.23, and negative experiences, *M* = 1.16, *SD* = 1.82, *ps* < .003. The difference between positive and negative experiences was not significant, *p* = .014. For Latino gangs, shared positive experiences was the main factor of fusion with the group, *M* = 4.56, *SD* = 1.94, as compared to shared values, *M* = 3.90, *SD* = 2.16, *p* = .008, but not compared to shared negative experiences, *M* = 4.32, *SD* = 2.16, *p* = .017. For delinquent bands, no significant differences were found for shared values, *M* = 2.07, *SD* = 2.36, shared positive experiences, *M* = 2.49, *SD* = 2.53, and shared negative experiences, *M* = 2.71, *SD* = 2.64, *ps* < .14. There was also a main effect of group, (*F*(3,223) = 21.90, *p* < .001, *η_p_^2^* = 0.23), and a main effect of factor, (*F*(1,223) = 8.07, *p* < .001, *η_p_^2^* = 0.04).

Additional analyses were conducted to determine which of the three potential factors of fusion with the group was more strongly associated with fusion with group at time of sentencing, and fusion with the group at present. For jihadists, shared important values was the only significant predictor for fusion at time of sentencing, *b* = 0.44, *p* = .007, and at present, *b* = 0.33, *p* = .05 (see Fig. 6). For Latino gangs, shared negative experiences was marginally associated with fusion with the group at time of sentencing, *b* = 0.23, *p* = .068, and shared positive experiences at present, *b* = 0.30, *p* = .045. For delinquent bands, shared values was the only factor significantly associated with fusion at time of sentencing, *b* = 0.54, *p* = .001, and at present, *b* = 0.46, *p* = .005. For non-jihadists, important values was the only factor signiticantly associated with fusion at time of sentencing, *b* = 0.28, *p* = .050, and at present, *b* = 0.37, *p* = .009.

*Perceived injustice in sentencing.* Univariate analyses on perceived injustice in sentencing yielded a significant effect, *F*(3,261) = 7.863, *p* < .001, *η_p_^2^* = .08). Jihadists perceive the highest injustice in sentencing, *M* = 3.27, *SD* = 2.19, compared to Latino gangs, *M* = 1.91, *SD* = 2.09, *p* = .002, bands, *M* = 2.09, *SD* = 2.26, *p* = .029, and non-jihadists, *M* = 1.56, *SD* = 2.15, *p* < .001. No differences were found between the other groups, *ps* > 0.91.

*Perceived Personal Discrimination.* Univariate analyses on perceived personal discrimination yielded a significant effect, *F*(3,237) = 12.846, *p* < .001, *η_p_^2^* = .14). Jihadists perceive more personal discrimination, *M* = 3.30, *SD* = 2.14, compared to bands, *M* = 2.17, *SD* = 2.38, *p* = .052, and non-jihadists, *M* = 1.36, *SD* = 1.77, *p* < .001. No differences were found with Latino gangs, *M* = 3.09, *SD* = 1.95, *p* = 1. Latino gangs perceive more personal discrimination than non-jihadists, *p* < .001.

**Jihadists expressed more sacrifices for values in prison because they perceived more injustice.** To test whether perceived injustice in prison mediates stronger willingness for costly sacrifices for values in prison among jihadists compared to Latino gangs and compared to the full sample of criminals, we conducted a bootstrapping test (5,000 boots, Model 4) with PROCESS*,* controlling for time spent in prison (in months). The indirect effect via perceived injustice was significant when comparing jihadists with Latino gangs, IE = 0.52, 95% CI: [0.13, 1.03], and when comparing Jihadists with the full criminal sample, IE = 0.31, 95% CI: [0.09, 0.61] (see Figs. S7a, S7b).

**Injustice was related to costly sacrifices in prison through fusion with values and fusion with the Nation´s Justice System.** To test whether perceived injustice increase willingness to sacrifice for values by increasing fusion with values, and if feelings of injustice reduce willingness to sacrifice for values by increasing fusion with the Nation´s Justice System, we conducted a pair of bootstrapping tests (5,000 boots, Model 81) with PROCESS, controlling for time spent in prison (in months).

The indirect effect of perceived injustice on sacrifices for values in prison through fusion with values was significant when comparing jihadists with Latino gangs IE = 0.20, 95% CI: [0.04, 0.43], and when comparing jihadists with the full criminal sample, IE = 0.09, 95% CI: [0.01, 0.21]. The indirect effect on sacrifices for values through fusion with Nation´s Justice System was significant when comparing jihadists with Latino gangs IE = 0.13, 95% CI: [0.02, 0.30], and when comparing jihadists with the full criminal sample, IE = 0.06, 95% CI: [0.01, 0.14] (see Figs. S8a, S8b).

**Jihadists reduce perception of injustice via increasing fusion with family, thus reducing personal discrimination.** To test whether perceived injustice decrease as a function of fusion with family and personal discrimination, we conducted a pair of bootstrapping tests (5,000 boots, Model 6) with PROCESS*,* controlling for the time spent in prison (in months). The indirect effect of the group on perception of injustice via fusion with family and personal discrimination was significant when comparing jihadists with Latino gangs IE = -0.20, 95% CI: [-0.38, 0.07], and when comparing jihadists with the full criminal sample, IE = -0.12, 95% CI: [-0.23, -0.05] (see Figs. S9a, S9b).

**References**

^1^ Atran, S., Axelrod, R. & Davis, R. Sacred barriers to conflict resolution. *Science* 317*,*1039-1040 (2007).

^2^ Gómez, Á. et al., Recent advances, misconceptions, untested assumptions, and future agenda for identity fusion theory. *Soc. Personal. Psychol. Compass* e12531 (2020).

^3^ Reinares, F., García-Calvo, C. & Vicente A. From criminals to terrorists and back. Quarterly Report Vol. 2: Spain; Real Instituto Elcano. *GLOBSEC* <https://www.globsec.org/publications/from-criminals-to-terrorists-and-back-quarterly-report-spain-vol-2/> (2019).

**Supplementary Figures**

Fig. S1. Spanish Prison Map including the Penitentiary Centers where interviews were conducted and the number of interviews per Center.

**Fig. S2.** The degree to which inmates considered sacrifices extreme while in prison (Preliminary Study).

Fig. S3. Percentage of jihadists versus others (non-jihadists + delinquent bands) citing at least one thematic confirmation for each of eight analytic categories.

Fig. S4. Means of thematic confirmations for jihadists versus Latino gangs.

Fig. S5. Means of thematic confirmations for jihadists versus others (non-jihadists + delinquent bands).

**Fig. S6**. Results of regression analyses illustrating that for jihadists, only shared values with the group predict fusion with the group at time of sentencing and fusion with the group at present.

**Fig. 7a.** Jihadists expressed greater willingness to make sacrifices for values in prison than Latino gangs because they perceive stronger feelings of injustice.

**Fig. 7b.** Jihadists expressed greater willingness to make sacrifices for values in prison than the full sample of criminals because they perceive stronger feelings of injustice.

**Fig. 8.** Full mediation analysis showing: 1) jihadists express more feelings of perceived injustice in sentencing than do all other convicts sampled; 2) their feelings of injustice increase willingness to sacrifice for values by increasing fusion with values; 3) lessening levels of perceived injustice increase fusion with Nation´s Justice System, which in turn reduces willingness to sacrifice for (religious) values.

**Fig. 9.** Full mediation analyses showing: 1) jihadists express more fusion with family and personal discrimination than do all other convicts sampled; 2) fusion with family reduces the perception of personal discrimination, which in turn is associated with less perceived injustice in sentencing.

**Supplementary Tables**

**Table S1.** Category framework for Content Analysis.

**Table S2**. Number and percentage of participants of each group who cited at least one thematic indicator related to each category.

**Table S3.** Within-group differences regarding the percentage of people citing thematic indicators of opposite categories. A significant positive McNemar test indicates that the positive pole (e.g., fusion) was cited more than the negative pole (e.g., no fusion).

**Table S4.** Comparisons between jihadists and gangs who cited at least one thematic indicator related to each category.

**Table S5.** Comparisons between jihadists and other groups who cited at least one thematic indicator related to each category.

**Table S6.** Numbers, means and standard deviations of thematic indicators for jihadists, Latino gangs and others.

**Table S7.** Within-group differences regarding the number of thematic indicators of opposite categories.

**Table S8.** Comparisons between jihadists and gangs regarding the number of thematic indicators related to each category.

**Table S9.** Comparisons between jihadists and other groups regarding the number of thematic indicators related to each category.

**Table S10.** Examples of the thematic indicators in favor or against fusion with the group.

**Table S11.** Examples of the thematic indicators in favor or against fusion with the value.

**Table S12.** Examples of the thematic indicators in favor or against perceptions of injustice.

**Table S13.** Examples of the thematic indicators in favor or against personal discrimination.

**Table S14.** Examples of the thematic indicators in favor or against fusion with the family.

**Table S15.** Examples of the thematic indicators in favor or against fusion with the Nation´s Justice System.

**Table S16.** Examples of the thematic indicators in favor or against sacrifices for the group.

**Table S17.** Examples of the thematic indicators in favor or against sacrifices for the value.

**Table S18.** Correlation between variables (full sample).

**Table S19.** Correlation between variables (jihadists and non-jihadist Muslims).

**Table S20.** Correlation between variables (Latino gangs and delinquent bands).

**Table S21.** Univariate Analyses for each of the outcome measures on each group (*F*s, Means and Standard Deviations).

**Supplementary Figures**

Fig. S1. Spanish Prison Map including the Penitentiary Centers where interviews were conducted and the number of interviews per Center.

**The map used as a basis to draw the image was taken from**  [**https://d-maps.com/carte.php?num_car=5672&lang=es**](https://d-maps.com/carte.php?num_car=5672&lang=es%E2%80%9D)

**Fig. S2. The degree to which inmates considered sacrifices extreme while in prison.** All sacrifices were significantly higher than the scale’s mid-point and reliably related (*α* = .71); however willingness to “die for someone or something“ was dropped from the final survey owing to the unease that some participants expressed in responding to the item.

Fig. S3. Percentage of jihadists versus others (non-jihadists + delinquent bands) citing at least one thematic confirmation for each of eight analytic categories.

Fig. S4. Means of thematic confirmations for jihadists versus Latino gangs.

Fig. S5. Means of thematic confirmations for jihadists versus others (non-jihadists + delinquent bands).

**Fig. S6. Results of regression analyses illustrating that for jihadists, only shared values with the group predict fusion with the group at time of sentencing and fusion with the group at present.**

**Fig. 7a. Jihadists expressed greater willingness to make sacrifices for values in prison than Latino gangs because they perceive stronger feelings of injustice** (based on a bootstrapping test (5,000 boots, Model 4) with PROCESS*;* *b* values are unstandardized β values; *p < .05, **p < .01, ***p < .001).

**Fig. 7b. Jihadists expressed greater willingness to make sacrifices for values in prison than the full sample of criminals because they perceive stronger feelings of injustice** (based on a bootstrapping test (5,000 boots, Model 4) with PROCESS; *b* values are unstandardized β values; ***p < .001).

**Fig. 8. Full mediation analysis showing that: 1) jihadists express more feelings of perceived injustice in sentencing than do all other convicts sampled; 2). their feelings of injustice increase willingness to sacrifice for values by increasing fusion with values; 3) lessening levels of perceived injustice increase fusion with Nation´s Justice System, which in turn reduces willingness to sacrifice for (religious) values** (based on a bootstrapping test (5,000 boots, Model 81) with PROCESS; *b* values are unstandardized β values; *p < .05, **p < .01, ***p < .001).

**Fig 9. Full mediation analysis showing that: 1) jihadists express more fusion with family and personal discrimination than do all other convicts sampled; 2) fusion with family reduces perception of personal discrimination, which in turn is associated with less perceived injustice in sentencing** (based on a bootstrapping test (5,000 boots, Model 6) with PROCESS; *b* values are unstandardized β values; *p < .05, **p < .01, ***p < .001).

**Supplementary Tables**

**Table S1. Category framework for Content Analysis (Thematic Indicators).**

| Category | Thematic Indicators | | | | |
| --- | --- | --- | --- | --- | --- |
| 1. **Fusion/No fusion with the reference group** | *Reference Group*: the group of “Muslims” for jihadists and non-jihadist Muslims, “the gang” for members of Latino gangs, and “the band” for delinquent bands. | | | | |
| **Fusion with the group.** The participant expresses positive feelings of union, belonging, affection, priority, approval, positive evaluations towards the reference group, or positive experiences with the group at any temporal moment (past, present, or future). | - Unity - Oneness - Connection - Like a family - Second family - Brotherhood - Brothers | | - Support - Understanding - Love - Affection - Good - Raised with - Friendship - Sharing | | - Trust - Not failed me - A reference to me - My people - Together - My own life - Impossible leaving the group |
| **No fusion with the group.** The participant expresses feelings of rejection, detachment, disapproval, abandonment, betrayal, incomprehension, negative evaluations towards the reference group, or negative experiences due to the group at any temporal moment (past, present, or future). | - No belonging - No attachment - No bonding - No support - No understanding - No ties - No relationship - No contact - Nothing to do with - Disconnection - Unlinked | | - Different from - Distance - Indifference - No interest - Do not matter - Do not care about - No trust - Betrayal - Do not share - Problems with | | - Falsity - Selfish - Profit-seeker - Traitors - Vermin - Dirty - Backward minded - Humiliation - Hurt/Harm - Bad treatment |
| 2. **Fusion/No fusion with the specific value** | *Value*: the value of “religion*”* for jihadists and non-jihadist Muslims, and the value of “honor” for Latino gangs and delinquent bands | | | | |
| **Fusion with the value.** The participant considers that the value is an essential part of him or an important guiding principle in his life, defends its true meaning, or conveys a strong commitment to the value at any temporal moment (past, present, or future). | - Religion/religious - Islam - God - Quran - Pray - Faith - Believer - Value(s) | - Total connection with the value - Practicing religion - Understanding the value - Honor - Sense of honor - Is important | | - True Muslim - Orthodox - Rectitude - Proud to be Muslim - God commands - I believe - Ramadan | |
| **No fusion with the value.** The participant expresses an explicit opposition to the value, disaffection, or indifference for it at any temporal moment (past, present, or future). | - Atheist - Non-religious - Far from religion - No praying | - No practicing - Believing in nothing | | - Nothing to do with the value - No interest in the value | |
| 3. **Perceived Injustice/Justice** |  | | | | |
| **Perception of justice.** The participant expresses feelings and perceptions of justice, fairness, legitimacy about his sentence, his personal situation,  or about the functioning of the justice system in general, at any temporal moment (past, present, or future). | - Fairness - Fair - Deserve | | - Guilt/Guilty - Committed a crime - I have to pay | | - Recognizing - Rehabilitation |
| **Perception of injustice.** The participant expresses feelings and perceptions of injustice, unfairness, illegitimacy about his sentence, his personal situation, or about the functioning of the justice system in general, at any temporal moment (past, present, or future). | - Unfairness/   Unfair/Unfairly   - Not deserving - Not justified | | - Lie - False - Not guilty - Innocent | | - I did nothing - No proofs - Victim - Unjust penalty |
| 4. **Perceived Personal Discrimination/**   **No discrimination** |  | | | | |
| **Discrimination.** The participant refers experiences of being treated badly due to a personal trait or to his group belonging at any temporal moment (past, present, or future). | - Prejudice - Stereotypes - Racism - Xenophobia - Discrimination - Abuse - Insulted - Cataloged - Labelled - Treated badly/   contemptuously   - Undervalued | | - Bullying - Critics - Suffering - Arbitrary judgement - Mocking - Rejection - Prototype - Humiliation - Persecution - Hatred - Uncomfortable - Witch-hunt | | - Meta-stereotypes - Terrorist - Ignorant - Radical - Monster - Enemy |
| **No discrimination.** The participant reports not being treated badly due to a personal trait or to his group belonging at any temporal moment (past, present, or future). | - No discrimination - Never discriminated | | - Valued - No oppression | | - Same opportunities |
| 5. **Fusion/No fusion with family** | Reference: Family, Parents/Mother/Father, Grandmother, Brother(s)/Sister(s)  Children (offspring), Son/Daughter | | | | |
| **Fusion with the family.** The participant expresses feelings of deep union, love, or belonging to his family at any temporal moment (past, present, or future). | - Union - Important - Love - Affection | | - Caring - Sense of life - Life - Happiness - Family support | | - Beautiful - Strength - Die for my family - Inseparable - Get along with |
| **No fusion with the family**. The participant expresses feelings of rejection or detachment from his family at any temporal moment (past, present, or future). | - No love - No sharing - No contact | | - Bad relationship - Lonely - Beating - Punishment | | - Marginalized - Oblivion - Abandonment |
| 6. **Fusion/No fusion with Nation’s Justice System** | Reference: Spain, Justice system, Prison staff, Prison, Police, Civil Guards, Forces and Bodies of the State Security | | | | |
| **Fusion with Nation’s Justice System.** The participant expresses positive feelings toward groups and values that represent the Nation’s Justice System, such as Spain, democracy, forces and bodies of the State Security, police, civil guard, and prison staff, or reports positive treatment by them at any temporal moment (past, present, or future). | - Good treatment - No complaints - Secure - Calm | | - Correct - Nice - Good people - Wonderful people | | - Listen - Help - Respect - Support |
| **No fusion with Nation’s Justice System.** The participant expresses negative feelings and evaluations towards groups and values that represent the Nation’s Justice System, such as Spain, democracy, forces, and bodies of the State Security police, civil guard, and prison staff, or negative treatment by them at any temporal moment (past, present, or future). | - Bad treatment - Treated like a dog - Fatal - Humiliation - Threats/Threaten - No relation - Control - Hate - Power abuse - Contempt - Ignored | | - Against the system - Corrupt/   mafioso   - Abuse - Mistreatment - Beating - Hitting - Danger - No trust - No empathy | | - Bastards - Authoritative people (fascists) - Cowards - Stupid - Bullies - Bad |
| 7. **Sacrifice/No sacrifice for the reference group** |  | | | | |
| **Sacrifice for the reference group.** The participant reports behaviors that imply personal sacrifices or strong dispositions to defend, protect, or improve the situation of the reference group. | - Willingness to renounce/renouncing - Defending - Surrendering - Helping - Supporting | | - Killing for - Bloodshed - Stabbing - Machete blows - Fighting - Stealing - Going to Syria | | - Fighting for your people - Proving one's self to become part of the group - Proving - Demonstrating |
| **No sacrifice for the reference group.** The participant refuses to make behaviors that imply personal sacrifices for the reference group or reports an explicit opposition to defend, protect, or improve the situation of the reference group. | - Nothing for anyone - No sense - Sacrificing nothing | | - No renounce for - No dying for | | - No defending - No going back to prison for |
| 8. **Sacrifice/No sacrifice for the specific value** |  | | | | |
| **Sacrifice for the specific value.** The participant reports behaviors that imply sacrifices or strong dispositions to defend or protect the specific value. | - Leaving everything behind for - Fighting for what I believe | | - Defending - Dying for - Killing for | | - Entering in prison again for - Complying with the value |
| **No sacrifice for the specific value.**  The participant refuses to make behaviors that imply personal sacrifices for the specific value or reports an explicit opposition to defend or protect it. | - No renouncing for - No defending | | - Never - No dying for | | - No going back to prison |

**Table S2. Number and percentage of participants of each group who cited at least one thematic indicator related to each category.**

|  | Jihadists  *N* = 50 | | Latino gangs  *N* = 69 | | Latino gangs + Non-jihadist + Delinquent bands  *N* = 190 | |
| --- | --- | --- | --- | --- | --- | --- |
| Category | *n* | % | *n* | % | *n* | % |
| Fusion with group | 22 | 44.0 | 44 | 63.8 | 77 | 40.5 |
| No fusion with group | 22 | 44.0 | 51 | 73.9 | 125 | 65.8 |
| Fusion with value | 28 | 56.0 | 16 | 23.2 | 50 | 26.3 |
| No fusion with value | 7 | 14.0 | 11 | 15.9 | 49 | 25.8 |
| Injustice | 49 | 98.0 | 28 | 40.6 | 102 | 53.7 |
| Justice | 3 | 6.0 | 41 | 59.4 | 95 | 50.0 |
| Personal discrimination | 34 | 68.0 | 29 | 42.0 | 69 | 36.3 |
| No Personal discrimination | 4 | 8.0 | 2 | 2.9 | 18 | 9.5 |
| Fusion with family | 29 | 58.0 | 51 | 73.9 | 133 | 70.0 |
| No fusion with family | 6 | 12.0 | 25 | 36.2 | 36 | 18.9 |
| Fusion with Nation´s Justice System | 17 | 34.0 | 26 | 37.7 | 66 | 34.7 |
| No fusion with Nation´s Justice System | 34 | 68.0 | 42 | 60.9 | 89 | 46.8 |
| Sacrifice for group | 8 | 16.0 | 28 | 40.6 | 31 | 16.3 |
| No sacrifice for group | 3 | 6.0 | 6 | 8.7 | 17 | 8.9 |
| Sacrifice for value | 10 | 20.0 | 10 | 14.5 | 14 | 7.4 |
| No sacrifice for value | 1 | 2.0 | 2 | 2.9 | 10 | 5.3 |

**Table S3. Within-group differences regarding the percentage of people citing thematic indicators of opposite categories***. A significant positive McNemar test indicates that the positive pole (e.g., fusion) was cited more than the negative pole (e.g., no fusion).*

|  | Jihadists  *N* = 50 | | Latino gangs  *N* = 69 | | Latino gangs + Non-jihadists + Delinquent bands  *N* = 190 | |
| --- | --- | --- | --- | --- | --- | --- |
| Category | *McNemar test* | *p* | *McNemar test* | *p* | *McNemar test* | *p* |
| Fusion – No fusion with group | 0.05 | .831 | 1.24 | .265 | 23.01 | <.001 |
| Fusion – No fusion with value | 12.90 | <.001 | 0.70 | .404 | 0.00 | 1.00 |
| Injustice – Justice | 44.02 | <.001 | 2.82 | .092 | 0.27 | .600 |
| Personal discrimination – No personal discrimination | 26.28 | <.001 | 23.31 | <.001 | 31.65 | <.001 |
| Fusion – No fusion with family | 15.61 | <.001 | 15.63 | <.001 | 76.17 | <.001 |
| Fusion – No fusion with justice | 8.83 | .003 | 7.03 | .008 | 5.09 | .024 |
| Sacrifice – No sacrifice for group | 1.45 | .228 | 15.75 | <.001 | 4.02 | .045 |
| Sacrifice – No sacrifice for value | 5.82 | .016 | 4.08 | .043 | 0.41 | .522 |

**Table S4. Comparisons between jihadists and gangs who cited at least one thematic indicator related to each category.**

| Category | *b* | *se* | Wald’s χ^2^ | *p* | Exp(*b*) | 95% CI | |
| --- | --- | --- | --- | --- | --- | --- | --- |
| Fusion with group | -0.81 | 0.38 | 4.52 | .034 | 0.45 | 0.21 | 0.94 |
| No fusion with group | -1.28 | 0.40 | 10.52 | .001 | 0.28 | 0.13 | 0.60 |
| Fusion with value | 1.44 | 0.40 | 12.74 | <.001 | 4.22 | 1.91 | 9.29 |
| No fusion with value | -0.15 | 0.52 | 0.09 | .771 | 0.86 | 0.31 | 2.40 |
| Injustice | 4.27 | 1.04 | 16.90 | <.001 | 71.75 | 9.35 | 550.33 |
| Justice | -3.13 | 0.64 | 23.67 | <.001 | 0.04 | 0.01 | 0.15 |
| Personal disc. | 1.08 | 0.39 | 7.64 | .006 | 2.93 | 1.37 | 6.28 |
| No personal disc. | 1.07 | 0.89 | 1.45 | .228 | 2.91 | 0.51 | 16.57 |
| Fusion with family | -0.72 | 0.40 | 3.28 | .070 | 0.49 | 0.22 | 1.06 |
| No fusion with family | -1.43 | 0.50 | 8.08 | .004 | 0.24 | 0.09 | 0.64 |
| Fusion with justice | -0.16 | 0.39 | 0.17 | .680 | 0.85 | 0.40 | 1.82 |
| No fusion with justice | 0.31 | 0.39 | 0.64 | .425 | 1.37 | 0.64 | 2.94 |
| Sacrifice for group | -1.28 | 0.46 | 7.80 | .005 | 0.28 | 0.11 | 0.68 |
| No sacrifice for group | -0.40 | 0.73 | 0.30 | .585 | 0.67 | 0.16 | 2.82 |
| Sacrifice for value | 0.39 | 0.49 | 0.62 | .429 | 1.48 | 0.56 | 3.87 |
| No sacrifice for value | -0.38 | 1.24 | 0.09 | .759 | 0.68 | 0.06 | 7.76 |

**Table S5. Comparisons between jihadists and other groups who cited at least one thematic indicator related to each category**

| Category | *b* | *se* | Wald’s χ^2^ | *p* | Exp(*b*) | 95% CI | |
| --- | --- | --- | --- | --- | --- | --- | --- |
| Fusion with group | 0.14 | 0.32 | 0.20 | .657 | 1.15 | 0.62 | 2.16 |
| No fusion with group | -0.90 | 0.32 | 7.66 | .006 | 0.41 | 0.22 | 0.77 |
| Fusion with value | 1.27 | 0.33 | 14.91 | <.001 | 3.56 | 1.87 | 6.79 |
| No fusion with value | -0.76 | 0.44 | 2.97 | .085 | 0.47 | 0.20 | 1.11 |
| Injustice | 3.74 | 1.02 | 13.46 | <.001 | 42.28 | 5.72 | 312.46 |
| Justice | -2.75 | 0.61 | 20.15 | <.001 | 0.06 | 0.02 | 0.21 |
| Personal disc. | 1.32 | 0.34 | 15.09 | <.001 | 3.73 | 1.92 | 7.24 |
| No personal disc. | -0.19 | 0.58 | 0.10 | .748 | 0.83 | 0.27 | 2.58 |
| Fusion with family | -0.53 | 0.33 | 2.57 | .109 | 0.59 | 0.31 | 1.12 |
| No fusion with family | -0.54 | 0.47 | 1.30 | .254 | 0.58 | 0.23 | 1.47 |
| Fusion with justice | -0.03 | 0.34 | 0.01 | .922 | 0.97 | 0.50 | 1.87 |
| No fusion with justice | 0.88 | 0.34 | 6.85 | .009 | 2.41 | 1.25 | 4.66 |
| Sacrifice for group | -0.02 | 0.43 | 0.00 | .957 | 0.98 | 0.42 | 2.28 |
| No sacrifice for group | -0.43 | 0.65 | 0.44 | .505 | 0.65 | 0.18 | 2.31 |
| Sacrifice for value | 1.15 | 0.45 | 6.49 | .011 | 3.14 | 1.30 | 7.59 |
| No sacrifice for value | -1.00 | 1.06 | 0.89 | .345 | 0.37 | 0.05 | 2.94 |

**Table S6. Numbers, means and standard deviation of thematic indicators for jihadists, Latino gangs and others.**

|  | Jihadists  *N* = 50 | | | Latino gangs  *N* = 69 | | | Latino gangs + Non-jihadists + Delinquent bands  *N* = 190 | | |
| --- | --- | --- | --- | --- | --- | --- | --- | --- | --- |
| Category | *n* | *M* | *SD* | *n* | *M* | *SD* | *n* | *M* | *SD* |
| Fusion with group | 51 | 1.02 | 1.58 | 131 | 1.90 | 2.55 | 180 | 0.95 | 1.80 |
| No fusion with group | 36 | 0.72 | 1.09 | 181 | 2.62 | 2.67 | 342 | 1.80 | 2.14 |
| Fusion with value | 86 | 1.72 | 2.53 | 25 | 0.36 | 0.79 | 87 | 0.46 | 0.95 |
| No fusion with value | 17 | 0.34 | 1.00 | 12 | 0.17 | 0.42 | 72 | 0.38 | ‘0.77 |
| Injustice | 200 | 4.00 | 2.78 | 67 | 0.97 | 1.58 | 221 | 1.16 | 1.61 |
| Justice | 3 | 0.06 | 0.24 | 82 | 1.19 | 1.37 | 170 | 0.89 | 1.13 |
| Personal disc. | 87 | 1.74 | 2.02 | 57 | 0.83 | 1.29 | 116 | 0.61 | 1.14 |
| No personal disc. | 9 | 0.18 | 0.69 | 2 | 0.03 | 0.17 | 24 | 0.13 | 0.43 |
| Fusion with family | 71 | 1.42 | 1.73 | 157 | 2.28 | 2.13 | 339 | 1.78 | 1.83 |
| No fusion with family | 13 | 0.26 | 0.92 | 54 | 0.78 | 1.45 | 74 | 0.39 | 1.05 |
| Fusion with justice | 25 | 0.50 | 0.81 | 34 | 0.49 | 0.78 | 115 | 0.61 | 1.09 |
| No fusion with justice | 93 | 1.86 | 1.92 | 129 | 1.87 | 2.83 | 237 | 1.23 | 2.28 |
| Sacrifice for group | 16 | 0.32 | 0.89 | 71 | 1.03 | 1.66 | 74 | 0.39 | 1.12 |
| No sacrifice for group | 3 | 0.06 | 0.24 | 9 | 0.13 | 0.48 | 20 | 0.11 | 0.37 |
| Sacrifice for value | 24 | 0.48 | 1.16 | 14 | 0.20 | 0.56 | 19 | 0.10 | 0.39 |
| No sacrifice for value | 1 | 0.02 | 0.14 | 3 | 0.04 | 0.27 | 12 | 0.06 | 0.28 |

**Table S7. Within-group differences regarding the number of thematic indicators of opposite categories.**

|  | Jihadists  *N* = 50 | | Latino gangs  *N* = 69 | | Latino gangs + Non-jihadists + Delinquent bands  *N* = 190 | |
| --- | --- | --- | --- | --- | --- | --- |
| Category | *z* | *p* | *z* | *p* | *z* | *p* |
| Fusion – No fusion with group | -1.17 | .241 | -1.91 | .057 | -5.22 | <.001 |
| Fusion – No fusion with value | -3.31 | .001 | -1.61 | .107 | -0.74 | .459 |
| Injustice – Justice | -6.05 | <.001 | -1.15 | .249 | -1.17 | .242 |
| Personal disc. – No personal disc. | -4.92 | <.001 | -4.62 | <.001 | -5.28 | <.001 |
| Fusion – No fusion with family | -3.70 | <.001 | -4.69 | <.001 | -8.49 | <.001 |
| Fusion – No fusion with justice | -3.96 | <.001 | -4.15 | <.001 | -3.22 | .001 |
| Sacrifice – No sacrifice for group | -2.05 | .041 | -3.94 | <.001 | -3.28 | .001 |
| Sacrifice – No sacrifice for value | -2.70 | .007 | -1.99 | .047 | -1.06 | .289 |

**Table S8. Comparisons between jihadists and gangs regarding the number of thematic indicators related to each category.**

| Category | *b* | *se* | Wald’s χ^2^ | *p* | Exp(*b*) | 95% CI | |
| --- | --- | --- | --- | --- | --- | --- | --- |
| Fusion with group | -0.62 | 0.17 | 14.17 | <.001 | 0.54 | 0.39 | 0.74 |
| No fusion with group | -1.29 | 0.18 | 50.19 | <.001 | 0.27 | 0.19 | 0.39 |
| Fusion with value | 1.56 | 0.23 | 46.99 | <.001 | 4.75 | 3.04 | 7.,41 |
| No fusion with value | 0.67 | 0.38 | 3.16 | .075 | 1.96 | 0.93 | 4.09 |
| Injustice | 1.42 | 0.14 | 100.59 | <.001 | 4.12 | 3.12 | 5.43 |
| Justice | -2.99 | 0.59 | 25.81 | <.001 | 0.05 | 0.02 | 0.16 |
| Personal disc. | 0.75 | 0.17 | 19.11 | <.001 | 2.11 | 1.51 | 2.94 |
| No personal disc. | 1.83 | 0.78 | 5.46 | .019 | 6.21 | 1.34 | 28.74 |
| Fusion with family | -0.47 | 0.14 | 10.87 | .001 | 0.62 | 0.47 | 0.83 |
| No fusion with family | -1.10 | 0.31 | 12.72 | <.001 | 0.33 | 0.18 | 0.61 |
| Fusion with justice | 0.15 | 0.26 | 0.00 | .956 | 1.02 | 0.61 | 1,70 |
| No fusion with justice | -0.01 | 0.14 | 0.00 | .970 | 1.00 | 0.76 | 1.30 |
| Sacrifice for group | -1.17 | 0.28 | 17.81 | <.001 | 0.31 | 0.18 | 0.54 |
| No sacrifice for group | -0.78 | 0.67 | 1.34 | .244 | 0.46 | 0.13 | 1.70 |
| Sacrifice for value | 0.86 | 0.34 | 6.56 | .010 | 2.37 | 1.22 | 4.57 |
| No sacrifice for value | -0.78 | 1.15 | 0.45 | .501 | 0.46 | 0.05 | 4.42 |

**Table S9. Comparisons between jihadists/other groups regarding the number of thematic indicators related to each category.**

| Category | *b* | *se* | Wald’s χ^2^ | *p* | Exp(*b*) | 95% CI | |
| --- | --- | --- | --- | --- | --- | --- | --- |
| Fusion with group | 0.07 | 0.16 | 0.22 | .641 | 1.08 | 0.79 | 1.47 |
| No fusion with group | -0.92 | 0.18 | 27.35 | <.001 | 0.40 | 0.28 | 0.56 |
| Fusion with value | 1.32 | 0.15 | 75.75 | <.001 | 3.76 | 2.79 | 5.06 |
| No fusion with value | -0.11 | 0.27 | 0.16 | .688 | 0.90 | 0.53 | 1.52 |
| Injustice | 1.24 | 0.10 | 160.17 | <.001 | 3.44 | 2.84 | 4.16 |
| Justice | -2.70 | 0.58 | 21.53 | <.001 | 0.07 | 0.02 | 0.21 |
| Personal disc. | 1.05 | 0.14 | 54.53 | <.001 | 2.85 | 2.16 | 3.76 |
| No personal disc. | 0.35 | 0.39 | 0.82 | .365 | 1.43 | 0.66 | 3.07 |
| Fusion with family | -0.23 | 0.13 | 3.06 | .080 | 0.80 | 0.62 | 1.03 |
| No fusion with family | -0.40 | 0.30 | 1.81 | .179 | 0.67 | 0.37 | 1.20 |
| Fusion with justice | -0.19 | 0.22 | 0.75 | .387 | 0.83 | 0.54 | 1.27 |
| No fusion with justice | 0.40 | 0.12 | 10.66 | .001 | 1.49 | 1.17 | 1.90 |
| Sacrifice for group | -0.20 | 0.28 | 0.51 | .476 | 0.82 | 0.48 | 1.41 |
| No sacrifice for group | -0.56 | 0.62 | 0.82 | .364 | 0.57 | 0.17 | 1.92 |
| Sacrifice for value | 1.57 | 0.31 | 26.09 | <.001 | 4.80 | 2.63 | 8.76 |
| No sacrifice for value | -1.15 | 1.04 | 1.22 | .269 | 0.317 | 0.04 | 2.44 |

**Table S10. Examples of the thematic indicators in favor or against fusion with the group.**

|  | Jihadist | Latino gangs | Non-jihadist + Delinquent bands |
| --- | --- | --- | --- |
| Examples in favor of fusion with the group | .- “Mis amigos musulmanes son como mi familia”  .- “Los musulmanes me acogieron” (converted to Islam)  .- “Los musulmanes siempre te apoyan”  .- “No he sentido miedo en la guerra porque estaba con mi gente” (retornee)  .- “En la guerra con mis compañeros comíamos juntos, vivíamos juntos, no había nada negativo” (retornee)  .- “Nada me haría estar menos unido a mi grupo”  .- “En la calle, no tenía ningún motivo para sentirme conectado con los musulmanes, en la prisión sí, que son más respetuosos, más educados, más personas” | .- “Me iría de ellos (de la banda de los Ñetas), pero mi corazón siempre va a ser Ñeta. ¿Entiendes? Mi corazón siempre va a ser Ñeta, en Francia, en Italia, en Cuba. Siempre va a ser Ñeta”  .- “Con todos tienes que demostrar amor. Unidad. Y ese amor y esa unidad hace la fuerza para nosotros. Nos une y nos da más fuerza para cualquier cosa”  .- “Hay un dicho que se dice: eres, fuiste y serás (siempre un miembro de la banda)”  .- “Porque ellos (miembros de la banda) me daban una… ¿cómo se dice? … protección,…”  .- “…el recibimiento … al yo ingresar en la banda fue muy bueno… fue muy cariñoso. Yo me sentí como si estuviera con toda mi familia, ¿sabes?”  .- “Porque mi segundo (es un líder de una banda) es como si fuera mi hermano, ¿sabes? Íbamos a muerte yo y él.  .- “Me sentía como… que éramos una sola persona… que éramos un grupo de personas que se hacía una, porque pensábamos igual, actuábamos igual…” | .- “Para mí todos somos iguales. Es como mi familia…. mi mujer, mis hijos, son todos mi familia. La familia es lo que te creas tú, no los que te impone la sociedad” (delinquent band)  .- “Yo los quería como hermanos, los quería más que a mi propia vida” (delinquent band)  .- “Me empecé a sentir conectado con los musulmanes porque eran hermanos en la práctica de adoración”  .- “Yo soy musulmán al 100% y quiero morir musulmán como mi familia”  .- “Tus paisanos, tus primeras novias … descubrir muchas cosas … con trece años empecé a descubrir esas cosas. ¡Yo tengo hijos con sus hermanas!. Un vínculo que después de treintaicinco años siguen estando ahí … Son tu familia, tu familia” (delinquent band) |
| Examples against fusion with the group | .- “El problema es que yo confío en Dios, no confío en los musulmanes”  .- “No todos los musulmanes que se llaman musulmanes, son de verdad musulmanes, porque hay mucha falsedad, muchos crímenes”  .- “Los musulmanes no están unidos”  .- “Mi relación con los musulmanes es muy distante”  .- “Me dan igual los musulmanes. Sus ideas políticas (que no las tienen) y su religión…” | .- “… desde que he entrado (en prisión), se han *olvidao* de mi”  .- “Yo ya no estoy ahí (en la banda) y no quiero estar ahí. Quiero salir. Ya no… no creo que me aportasen ya la misma felicidad que antes. Ya no… sería como una relación rota”  .- “No se han *preocupao* por mi familia, por mí. Y siguen con lo mismo, no evolucionan”  .- “Yo se lo digo a todos los Latin King que pasan por enfrente mía: sois una puta vergüenza para lo que lleváis y representáis. Para mí es así y lo siento así”  .- “Cuando vi que olvidaban a mi mejor amigo, que creció dentro de la banda, se ganó su respeto. Era muy tranquilo, nunca había tenido problemas con nadie. Lo mataron ¿Quién … se acuerda de él? (…) Nos criamos y crecimos juntos. Vi que nadie se acordaba de él y a mí eso …” | .- “. Yo creía que eran mis amigos y son alimañas. Me han robado, me han dado de lado” (delinquent band)  .- “Yo es que me he criado aquí, la verdad. Yo nunca he sido árabe”  .- “El ‘muslim’ está en el corazón, no con ellos. Yo soy diferente de ellos”  .- “Era trabajo y punto. El grupo me da igual”  .- “Yo no tengo nada que ver con ellos” (con los Musulmanes)  “Los musulmanes ni me entienden, ni me apoyan”  “Hay muchos musulmanes diferentes; hay gente que no piensa como yo, racistas, sucios; los jóvenes y los mayores son diferentes”  “Los musulmanes de aquí no me gustan. Fuman y me tratan mal” |

**Table S11. Examples of the thematic indicators in favor or against fusion with the value.**

|  | Jihadist | Latino gangs | Non-jihadist + Delinquent bands |
| --- | --- | --- | --- |
| Examples in favor of fusion with the value | .- “El Islam está por encima de mi mujer, mi casa, mi madre y de mi persona”  .- “He elegido una forma de vida. El Islam es una forma de vida”  .- “Los valores como persona, esto fue lo que me llamó la atención del Islam desde el principio”  .- “Esos valores (lo del Islam) para mí son importantes, porque esos valores son los que hacen una persona”  .- “Por curiosidad, me puse a leer el Corán… me quedé en shock. Me di cuenta de que siempre había buscado algo y que lo había encontrado. A partir de entonces sabía que mi vida había cambiado”  .- “Es mi religión, soy musulmán, y estoy contento y orgulloso de ello” | .- “La fama, el honor y el respeto que quisimos ganar, ya lo ganamos en su día. Todas esas muertes ya las hicimos nosotros (los Latin King) en el año 2000/2001. Entonces, el respeto ya lo ganamos”  .- “Siempre he sido una persona de palabra. Si yo doy mi palabra, tengo que cumplirla”.  .- “Poder, honor y lealtad”, estos son los slogans de mi banda (Black Panter).  .- “¿Pa’ qué la banda si no hay honor?”  .- “Claro que hay que hacer honor a tus colores” (Latin King)  .- “Los valores, los principios, las ideas; esas son las razones por las que soy de la banda.”  .- “Estoy muy orgulloso del honor de mi banda” | .- “Todo se basa en el honor y en el respeto” […]  .- “El honor siempre ha sido lo primero”  .- “Éramos gente de honor, sí” (delinquent band)  .- “La palabra de un grupo o de una amistad está por encima de la ley”  .- “El respeto se gana con fuerza” (delinquent band)  .- “Yo antes no estaba volcado en la religión, estaba todo el día de fiesta para aquí y para allá. Y como dicen, entrar en la cárcel te cambia. Y ahora sí me siento musulmán de verdad”  .- “Yo quiero Dios, tengo fe, soy creyente o practicante también, soy musulmán (…)” |
| Examples against fusion with the value | .- “A mí, si alguien me dice ‘Dios existe’, pues que venga y hable conmigo. A mí no me dijo nada Dios”  .- “Yo del Islam no tengo mucho conocimiento, y me da igual”  - “Yo no soy creyente ni nada de eso”  .- “No me interesan las religiones”  .- “Yo no soy religioso”  .- “Yo no me he convertido nunca al Islam ni a nada” | .- “Para mi grupo el honor no era importante. Nos repartíamos el dinero y ya”  .- “Me vale verga” (The participant uses a slangsentence when replying to how he felt about the Honor of his gang, which means “It is nothing for me”)  .- “Yo no siento honor por ella (la banda). En su momento sí podía sentir honor por ella, pero yo ahora mismo quiero ser una persona normal”  .- “¿Te inculcaban honor? Sí, te inculcaban honor, te inculcan grupo, pero que… después te da cuenta de que nada es así”  .- “La banda no era lo que decía. El honor tampoco era lo que decía. Y yo era una falsedad, como un dibujito. Porque yo hacía las cosas que ellos me decían en el momento. Vamos que yo pienso que todo eso era mentira” | .- “No me interesan, ¿entiendes?, nunca he querido saber nada del Islam. A mí me da igual, ¿me entiendes?”  .- “No puedes defender el honor. Los tres (members of band) somos unos … que no merecemos nada”  .- “I don’t care about Islam”  .- “Hay algo que se llama Sharia. Nosotros no lo seguimos”  .- “Nosotros no tenemos unos valores, como las bandas latinas (…) A lo mejor te llevabas bien con alguno porque también tenía niños y podías salir a comer, pero nada más” (delinquent band)  .- “Lo que nos unía era el dinero, nada más” (delinquent band) |

**Table S12. Examples of the thematic indicators in favor or against perceptions of injustice.**

|  | Jihadist | Latino gangs | Non-jihadist + Delinquent bands |
| --- | --- | --- | --- |
| Examples in favor of perception of injustice | .- “Por conocer a cierta gente uno acaba aquí, pero desgraciadamente injustamente y sin tener que ver nada”  .- “Hemos tenido (varios detenidos de un grupo) un juicio injusto”  .- “Esto me parece demasiado injusto y demasiado inhumano”  .- “A mí me detuvieron por captación. Pero nadie se presentó en el juicio a decir ‘esta persona me ha captado’”  .- “El 80 por ciento de la gente en prisión por terrorismo es inocente”  .- “Voy a un juicio sin siquiera saber de lo que me están acusando para poder defenderme”  .- “Tener videos violentos en el móvil no es para entrar en la cárcel”  .- “Yo no entiendo todavía de qué se me ha acusado; ni siquiera mi abogado se entera” | .- “Dijeron que yo le di a uno una puñalada y que yo le di la orden a dos más pa’ que lo apuñalaran, cuando eso es incierto”.  .- “Era totalmente mentira...yo no hice nada”  .- “Me han *condenao*, ¡pero vaya!, que no lo he hecho”  .- “… porque encima estoy aquí por un delito que ni he cometido yo”  .- “Estoy injustamente, la verdad. Porque yo no hice nada. A mí me están culpando por ser del barrio”  .- “Yo me considero inocente. Yo no he tenido nada que ver con eso. Aparece alguien que le apuñalan y nadie sabe quién ha sido. Estamos presos todos los que él (un testigo) dice que hemos sido”  .- “Tengo mucha ira dentro, claro, estoy aquí sin hacer *ná* de lo que me acusan” | .- “Me tratan injustamente hasta en prisión. En vez de dejarme salir a la escuela, por ejemplo, que es un derecho que tenemos a la educación, pues directamente te dice que por seguridad, no”  .- “Al meterme tanta alarma social, directamente ¿qué es lo que hacen? Me ponen como si fuera más peligroso y me ponen un control directo” (delinquent band talking about the treatment into prison)  .- “Estar aquí… no me la merezco” (Muslim)  .- “Me condenaron por la cara” (Muslim)  .- “Se están marcando conmigo un abuso importante” (Muslim)  .- “Lo malo es que te condenan por algo que no has hecho” (Muslim)  .- “Me parece injusto el tiempo que me han echado” (Muslim) |
| Examples against of perception of injustice | .- “A lo mejor hice cosas que no debería hacer, ver cosas que no debería ver,…”  .- “Lo estoy pagando porque sí lo he hecho”  .- “Si estoy aquí es por algo. Yo he intentado hacer la mayor autocrítica posible”  .- “Si estoy aquí porque soy radical pues vale”. | .- “He hecho algo y hay que pagar por ello”  .- “Yo estoy aquí por tonto. Porque me dieron la oportunidad de ser libre y no lo aproveché. No supe echar el ancla y…”  .- “Porque estoy pagando por algo, ¿no? lo que estuve haciendo”  .- “Estoy en prisión por no hacer las cosas bien, con cabeza”  .- “A lo hecho pecho y *pa’lante*” (talking about the sentence)  .- “Hablando con la verdad, fue justo. Porque tampoco nadie me mandó hacer eso” | .- “Creo que estoy en prisión porque es justo” (Muslim)  .- “Esto (his sentence) me lo merezco” (delinquent band)  .- “Como dicen los españoles, ‘quien la hace la paga’ (…) no me parece injusta” (Muslim)  .- “Yo me he ganado la sentencia; si hubiese estado trabajado en el campo, no estaría aquí” (Muslim)  .- “Yo sé que he cometido un delito que tengo que pagar, y eso es lo que hay” (Muslim)  .- “Es justa, perfectamente. Lo que estoy pagando está muy bien. Me merezco estar aquí” (Muslim)  .- “La condena que estoy cumpliendo me parece justa” (delinquent band)  .- “Yo tengo que asumir mi responsabilidad” (delinquent band)  .- “Cometí un delito, pues tendré que pagarlo” (delinquent band) |

**Table S13. Examples of the thematic indicators in favor or against personal discrimination.**

|  | Jihadist | Latino gangs | Non-jihadist + Delinquent bands |
| --- | --- | --- | --- |
| Examples in favor of personal discrimination | .- “Una cosa que me molesta mucho es que la gente piense que porque yo practique mi religión o porque me gusta mi religión piense que yo soy un radical… Por ejemplo, me he dejado la barba y la gente piensa que mientras no me quite la barba voy a seguir siendo un radical”  .- Yo estoy aquí por ser marroquí, porque los culpables que hicieron eso eran musulmanes, y yo soy marroquí. Si fuera otro, yo no estaría en la cárcel”  .- “Sí que me han discriminado por ser musulmán”  .- “En la cárcel me discriminan por lo que estoy” (His sentence as jihadist)  .- “Yo estoy normal aquí y me empiezan a perseguir porque soy musulmán y estoy con esas personas” (Other Muslims)  .- “Yo he sufrido muchísimo en mi vida porque me han discriminado” | .- “Cuando llegué a este país sufrí racismo"  .- “En el instituto había algunos que me trataban despectivamente: me llaman ‘negro’ y demás”  .- “En la calle me han dicho: ‘negro de mierda, vete a tu país’"  .- “Me he sentido discriminado por ser como soy”  .- “He ido a buscar trabajo a sitios y me han dicho que no por ser de banda”  .- “No quise en su momento llamarlo racismo porque no lo entendía (tenía unos 12/13 años), pero era eso”  .- “La policía me decía ‘puto negro’ y *tó*”  .- “Y los policías me dicen… ‘puto negro, vete a tu país’… racismo y todo eso… yo no lo veo bien” | .- “Son racistas y ya está” (Muslim)  .- “No en todo, pero sí. He sido víctima de eso de discriminación” (Muslim)  .- “Sí (…) soy una víctima por ser musulmán por los tiempos que corren”  .- “Me sentí discriminado por ser musulmán en el colegio”  .- “Hay gente que mira a los musulmanes y piensa que su religión les dice que tienen que hacer cosas malas, y como yo soy musulmán…”  .- “Cuando pasó el atentado en Barcelona, la gente me miraba como si fuera un Talibán”  .- “La gente piensa que si eres musulmán, eres terrorista”  .- “A mí tienen aquí por ser colombiano, por estar cerca de donde fue eso, por esas dos cosas, y me discriminan” (delinquent band) |
| Examples against of personal discrimination | .- “Nunca he sentido discriminación por ser musulmán”  .- “Yo rezo en la celda, no rezo en el patio; no me molestan por eso” (inside prison)  .- “No, a mí no me ponen ninguna pega cuando rezo (…) Ni por los libros religiosos, ni por el Corán que tengo. No, nunca me han puesto problemas por eso…” | .- “Yo he tenido las mismas oportunidades que un chaval cualquiera. Otra cosa es que a lo mejor *haiga* ido a otro sitio y por el hecho de que ya la gente sepa que soy de una banda hayan dicho: ‘oh, ese chaval es de una banda, mejor… mejor lo discriminamos o’… pero no, no me siento así”  .- “Da igual de donde seas, hay gente que te ayuda y gente que no. Yo no he tenido problemas” | .- “Pues a mí me hacen como a los españoles, me tratan igual, no tengo problemas” (Muslim talking about the treatment into prison)  .- “Yo soy musulmán y no puedo desear eso porque a mí, que he vivido con los cristianos y a mí nadie me ha hecho daño, al revés, me acuerdo (…) me traían leche y pan a la puerta cuando llegué al pueblo ese. Vivía en una casa abandonada y me trataban bien”  .- “Los cristianos me han dado trabajo, me han dado amor, me han llevado al hospital cuando he estado malo”  .- “No, no he sufrido ninguna discriminación” |

**Table S14. Examples of the thematic indicators in favor or against fusion with the family.**

|  | Jihadist | Latino gangs | Non-jihadist + Delinquent bands |
| --- | --- | --- | --- |
| Examples in favor of fusion with the family | .- “Yo no cambio (mi familia) por nada. Eso no tiene precio”  .- “Mis padres…. Y mi hermana es lo más *preciable* que tengo”  .- “Yo quiero a mi familia, mi padre, mi madre, mucho, mucho”  .- “Lo que más echo de menos es a la familia”  .- “Lo que más me importa son mis hijos y mi familia”  .- “Cuando pienso en mi familia me olvido del resto de cosas”  .- “Soy una persona muy apegada a mi familia” | .- “Mi familia es la única cosa que me puede hacer feliz”  .- “Mi mamá es sagrada”  .-“Porque *pa*’ mí mi madre ha sido algo muy grande porque ha *estao* cuando mi padre no ha *estao*”  .- “Primero está la familia”  .- “Mi abuela, la que fallece, es con la que estaba más *apegao* porque me cuidaba de niño… Había tenido mucho vínculo con ella”  .- “Porque yo a mi madre era… muy, muy… estábamos muy unidos” | .- “Mi padre fue mi mejor amigo, ¿vale? Mi padre me enseñó muchísimas cosas en la vida, me enseñó valores, principios. Entonces, yo también era su mejor amigo”  .- “Mis hijos son mi vida… son los dueños de mi vida”  .- “Lo más importante, mi mujer y familia, que siempre estoy pensando en ellos”  .- “Moriría por mi familia”  .-“Mi objetivo para mi es mi hijo, nada más. Ese es el objetivo que tengo”  .- “Mi mujer y mis hijos es lo que más me importa”  .- “A mí me importa mi familia. Para mí, mi familia es mi vida” |
| Examples against of fusion with the family | .- “La familia no me importa, ni siquiera saben que estoy en prisión”  .- “Pase de estar con mi abuela con mucho cariño y cuidado a estar con mi madre, que estaba todo el día trabajando…yo estaba todo el día solo. Es como no tener familia”  .- “Mi padre se fue a América cuando tenía 3 años. Como si no hubiera tenido”  .- “Mi madre es una persona muy difícil de entender, ¡qué quieres que te diga!…”  .- “Tuve falta de cariño maternal. Mi madre era una mujer muy fría”  .- “No sé nada de mi familia desde que entré en prisión”  .- “En prisión, mi familia no me ayuda nada, no tenemos relación” | .- “No lo quiero. Es que lo odio a muerte. Lo odio con todas mis fuerzas” (to his father)  .- “Aunque sufra, pero ese hombre tiene que estar bajo tierra. Tiene que estar muerto” (his father)  .- “Fue como un… un recibimiento muy frío. Entonces, yo estaba, yo estaba… como te digo…, yo estaba muy dolido, porque tenía 10 años, 9 años, que no veía a mi padre” (Talking about the meetting with his father and his stepbrohters, after his father leaved his mother)  .- “Mi padre se separó de mi madre cuando yo era un bebé y poco más. No he compartido con él mucho”  .- “Tengo hermanos, pero con ellos no me llevo bien” | .- “Como pagué un homicidio, mi madre como que se sentía avergonzada, ¿vale? ¿Qué pasa? Que ya mi madre olvidó todo el sacrificio que yo hice en mi juventud por ella y por mi padre”  .- “Les mandé a … y no quiero saber más nada de ellos. Mi hermano se hizo policía nacional, ¿vale? Ósea, enemigos totales”  .- “No he tenido ese afecto hacia ella. Ese amor que creo que le tendría cualquier hijo a su madre”  .- “Me siento alejado porque he pasado muchos años alejado de ellos” (to his family)  .- “Sí que tengo familia, pero no me veo mucho con ellos, ni me importa” |

**Table S15. Examples of the thematic indicators in favor or against fusion with Nation´s Justice System.**

|  | Jihadist | Latino gangs | Non-jihadist + Delinquent bands |
| --- | --- | --- | --- |
| Examples in favor of fusion with Nation´s Justice System | .- “Estoy en módulo de respeto de primer grado que es mejor que el aislamiento total, por lo que estoy muy agradecido”  .- “Valoro que la gente que te trata aquí esté bien preparada”  .- “Con los funcionarios de prisiones me llevo bien, me tratan bien”  .- “Yo he tenido muy buen trato aquí dentro de prisión, no me puedo quejar”  .- “Este país me ha dado mucho, mucho. La verdad es que no tengo ninguna queja” | .- “Con los funcionarios me llevo muy bien. Con todos”.  .- “Aquí hay muchos profesionales que te ayudan a no volver a delinquir”  .- “Los funcionarios hacen bien su papel”  .- “Policías: bien…me cogieron porque estaban haciendo su trabajo”  .- “La prisión me está viniendo bien".  .- A mí los que me han *ayudao* de verdad son los psicólogos y ya está. Me han *enseñao* a razonar, a hablar, a que salga de otra manera. Y a tener paciencia y ser más tranquilo. No buscar peleas”  .- “Están haciendo bien las cosas” (referring to forces, and bodies of the State Security police)  .- Aquí los funcionarios son respetuosos. No son, por así decirlo, la palabra, unos …. Son personas como yo y como tú” | .- “A mí me tratan muy bien aunque sea musulmán, yo hago mis cosas en el *chabolo*, me ven rezando en el *chabolo* y no pasa nada”  .- “Yo me siento dentro de España, me siento bien, no tengo problema”  .- “Los funcionarios de prisiones: me he hecho amigo de más de uno”  .- “Yo me gusta mucho, mucho España ... Me han tratado bien”  .- “Quieren que nos hagamos reaccionar; nos dan la oportunidad de hacer cosas” (talking about the Justice System) |
| Examples against of fusion with Nation´s Justice System | .- “Para cachearte te hacen desnudarte…eso es humillante. Me siento violado”  .- “El abogado, mentiroso; fiscal, mentiroso; juez, mentiroso; subdirector de seguridad aquí, gran mentiroso; directora, mentirosa. Todos mentirosos. Tengo pruebas que esta gente es racista. Mentirosos”  .- “Me siento despreciado por los funcionarios. Para ellos no tengo ningún valor”  .- “Te tratan como un perro”  .- “Los españoles ¿qué quieren?, ¿quieren dormir en paz o que la gente no sea musulmana? Es decir, que en España no se lucha contra el terrorismo, sino contra los musulmanes”  .- “Yo no veo el culpable éste solamente, el que está haciendo el atentado. Sí, es culpable porque va a matar inocentes. Pero el que le ha hecho eso, es este gobierno, este Estado. Entonces, hay que luchar. Es que esto es una guerra sucia” | .- “Los psicólogos de la cárcel no te ayudan. El psicólogo de la cárcel a mí me hizo mucho daño. Mucho daño”  .- “Los odio a muerte. Te lo juro. Me han hecho pensar que hay veces que no hacen bien su trabajo”  .- “Por mi causa, por mi delito he sido un poco *ignorao* por las distintas cárceles en las que he *estao* y entonces, sí, un poco *ignorao*”  .- “Hay muchas humillaciones, pero depende de la cárcel en la que estés y… los funcionarios ¿me entiendes? He sido *humillao.* Yo estaba en un *chabolo* y delante de los funcionarios me han *desnudao* y me han *pegao* y *to*”  .- “Son personas que no saben tratar contigo. Son desagradables, ¿sabes por qué? Porque cuanto tú no entras al juego que ellos quieren, no le vales”  .- “Fuerza física sí que tienen, pero porque son animales”, (when talking to forces, and bodies of the State Security police)  .- “¿Funcionarios de prisiones? Los odio”  .- “A los funcionarios les deberían hacer test psicológicos cada mes. (…) Son malos.” | .- “Son despiadados, siembran odio y vamos a acabar mal, no tienen ningún valor, ni como personas”  .- “Regular (..) por como entraron en mi casa cuando me detuvieron. Si ellos no me entienden, yo no puedo entender”  .- “Para los funcionarios y así somos unos números. Hay funcionarios buenos y funcionarios malos, pero bueno”  .-“En la condena pasada, por ejemplo, hubo funcionarios que me, ósea que me dieron bofetadas y todo. Y como quién dice sin hacer nada, por culpa de un compañero”  .- “Al director de seguridad lo denuncié quince veces”  .- “Siento odio por la policía española” |

**Table S16. Examples of the thematic indicators in favor or against sacrifices for the group.**

|  | Jihadist | Latino gangs | Non-jihadist + Delinquent bands |
| --- | --- | --- | --- |
| Examples in favor of sacrifices for the group | .- “Ir a Siria es la cosa más bonita que he hecho en mi vida. Ir a luchar por tu gente, por tu familia. Todos necesitan ayuda”.  .- “Luchaba contra el régimen del dictador y de su padre que mató a 40.000 personas en 7 días. Mi padre luchó entonces y 23 años después ha luchado su hijo”  .- “Me hice de una “ONG” para ir a Siria a ayudar a los musulmanes que estaban sufriendo”  .- “Estaba dispuesto a matar para proteger a los civiles de Siria cuando estuve allí”  .- “Derramaría mi sangre por un musulmán”  .- “La conexión que sentía con los musulmanes me hacía que sintiera la necesidad de ir a Siria ayudar a los musulmanes”  .- “No me importa mi vida, me importan los musulmanes. Si un musulmán está bien, a mí no me importa que yo esté mal” | .- “Yo si he estudiado y he hecho mucho deporte es por, como quien decir, los Latin Kings. Porque es el propósito, que estudiemos y que entrenemos. Entonces, yo si tengo estudios y eso se puede decir que es por los Latin King. Si he dejado de drogarme y eso es por ellos. Me he sacrificado por ellos”  .- “¿Que nos toca matarnos entre nosotros? Pues, como te he dicho, a lo mejor hoy me matan a un amigo y a lo mejor yo mañana salgo a matar a un amigo de ellos”  .- “Tienes que derramar sangre para demostrar de que tú quieres estar dentro (de la banda) porque es como se demuestra de que si tú realmente quieres estar dentro de ahí”.  .- Si hay que machetear a alguien por el grupo, pues se le machetea”  .- “Cuando ven tu estás siempre *pa*  eso, *pa* eso, *pa* eso, *pa*eso, *pa’* eso… ya pasas a guerrero… dar tus primeros pasos, ¿sabes?... con ellos y *to* eso”  .- “Yo no me lo pensaba dos veces. (…) Uno me dice: ‘¡Ey Diablo!’ (his nickname). Me dice: ‘¿Ves a esa mujer? Hazla un hijo.’ Voy. Sin miedo. Lo hago y ya está” | .- “Yo… si había que hacer algo, se hacía. (…) Eran como una mini-familia.  .- Eran (mi grupo) todo lo que me había faltado en la vida: ser reconocido (…) en el sentido de, joder, tienes un hermano. ¿Que no tienes dónde dormir? Vamos a mi casa. Aunque mi vieja nos eche a los dos a la calle, los dos vamos a la calle. Yo paso esa noche frío contigo porque tú no vas a pasar frío solo. Te he sacado a escondidas un tupper de comida para que puedas comer en el parque, porque sé que llevas días sin comer” (delinquent band)  .- “Estaría dispuesto a renunciar al dinero pero para defender a musulmanes (youngs Muslim in prison) que no tienen con qué defenderse, que no saben hablar bien español, no tienen medios, etc.” |
| Examples against of sacrifices for the group | .- “Si defiendo a los musulmanes perdiendo mi trabajo, ya estoy jodiendo a otro musulmán, mi mujer, mi hijo, no merece la pena”  .- “Ya está, ya no puedo defender a los musulmanes. Ahora tengo que defenderme a mí”  .- “Renunciaría a mis ingresos por mi familia, no por la banda” (“banda” refers to “jihadist group”) | .- “Ahora mismo, si el otro se buscó problemas, tienes que ir a pegar una puñalada a alguien. Y vas a la cárcel por los problemas del otro. A mí eso no… No le encuentro sentido”  .- “No, no volvería a hacer nada por la banda, porque ya me lo han dicho, y ni ….. volver a prisión por ellos, ni …. Y morir menos. Ya lo he hecho” | .- “Yo no haría nada por la banda”  .- “Yo no hago nada por ellos” (Muslims)  .- “No haría nada por ellos. Si tienen un problema, que lo resuelvan ellos” (Muslims)  .- “Yo no sacrifico nada por el grupo” (for his delinquent band)  .- “Yo no haría nada por nadie del grupo"(for his delinquent band) |

**Table S17. Examples of the thematic indicators in favor or against sacrifices for the value.**

|  | Jihadist | Latino gangs | Non-jihadist + Delinquent bands |
| --- | --- | --- | --- |
| Examples in favor of sacrifices for the value | .- “Lo haces , te sacrificas porque eres musulmán, y tu religión te dice que tienes que ayudar a toda la gente, indiferentemente que sea de tu religión o de otra”  .- “¿No defender mi religión? Pues sí, me sentiría culpable, claro. Tengo que hacer lo que sea, lo que haga falta”  .- “Moriría por mis ideas. Ya sabes, ¿no?”  .- “No me importa que los demás lo sepan. Lo que sea por mi religión. Me defendía, me ponía bien. Rezaba en los patios… Entonces, me ponía a rezar en los patios como diciendo de lo que me habéis *abusao*”  .- “Luchar por lo que creo es la mayor felicidad en mi vida”  .- “Sí, puedo hacer todo por mi religión. Lo que sea”  .- “Dejaría todo por mi religión.”  .- “No te puedo decir lo que haría por mi religión. Tú ya lo sabes, pero no te lo puedo decir. Sabes por qué, ¿verdad?”  .- “¿Que si moriría por mi religión? Tú ya sabes mi respuesta y ya sabes que no te lo puedo decir”. | .- “Siempre he tenido que ganarme el respeto a base de peleas […] Soy muy respetado, pero he tenido que ganármelo. Es cuestión de honor”  .- “Si te doy mi palabra, te la tengo que cumplir. Me cueste lo que me cueste. Aunque me cueste la vida. Te la cumplo”  .- “Si hoy muere uno de nosotros, al día siguiente tiene que morir uno de los tuyos sí o sí. Aunque sea en Cuatro Caminos, nos metemos en Cuatro Caminos, nos metemos en donde sea. Pero uno de los tuyos tiene que morir, aunque sea un niño. Siempre va a haber guerra” (gangs from Madrid)  .- “Para hacer la paz tienes que hacer la guerra. Es así. Nuestros valores”  .- “No quiero problemas, pero… ¿qué hago?, ¿los dejo que me roben, que…?, el próximo ¿que me ponga a cuatro patas y que me …? Es que… uno se tiene que hacer un hombre *pa* subsistir, la verdad. Un hombre quiere decir… que me respeten, que soy un hombre de honor” | .- “Defenderé al Islam, pero no a los musulmanes”  .- “¿Moriría por mi religión? Pues sí, en el sentido de son muchas cosas, por ejemplo, si vienes a la mezquita y quieres hacer lo que sea, pues lo haces”  .- “¿Qué si moriría yo por defender el Islam? Moriría, pero no mataría”  .- “El respeto y el honor se ganan con fuerza”  .- “Pues sí que la defendería (mi religion), la verdad. Haría lo que hiciera falta”  .- “Me voy a la Meca en el futuro, y lo que haga falta por mi religión” |
| Examples against of sacrifices for the value | .- “Yo creo que no hace falta nada para defender la religión. La religión se defiende a sí misma” | .- “Es que yo no tengo que demostrar nada a nadie por el honor”  .- “Si es por el honor, no tengo que demostrarle nada a nadie” | .- “Mi religión, yo no puedo defenderla. No la puedo defender”  .- “El tiempo de las Cruzadas ha terminado”  .- No. La vida es sagrada. Más que el honor o que cualquier cosa” |

**Table S18. Correlation between critical variables. Full sample**

| FULL SAMPLE | 2 | 3 | 4 | 5 | 6 | 7 | 8 | 9 | 10 | 11 | 12 | 13 |
| --- | --- | --- | --- | --- | --- | --- | --- | --- | --- | --- | --- | --- |
| 1. Fusion with value at Time of Sentencing | .53^***^  (*N*=250) | .40^***^  (*N*=249) | .26^***^  (*N*=249) | -.01  (*N*=230) | -.11  (*N=*204) | .14^*^  (*N=*244) | .27^***^  (*N*=229) | .32^***^  (*N*=217) | .35^***^  (*N*=216) | .44^***^  (*N*=227) | .40^***^  (*N*=220) | .27^***^  (*N*=215) |
| 2. Fusion with Value Now |  | .28^***^  (*N*=246) | .58^***^  (*N*=247) | .07  (*N*=277) | -.05  (*N*=202) | .25^***^  (*N*=241) | .24^***^  (*N*=227) | .40^***^  (*N*=216) | .51^***^  (*N*=216) | .54^***^  (*N*=225) | .27^***^  (*N*=218) | .10  (*N*=213) |
| 3. Fusion with Group at Time of Sentencing |  |  | .45^***^  (*N*=262) | -.02  (*N*=240) | -.04  (*N=*214) | .05  (*N*=254) | .26^***^  (*N*=230) | .33^***^  (*N*=216) | .24^***^  (*N*=213) | .46^***^  (*N*=235) | .44^***^  (*N*=228) | .32^***^  (*N*=223) |
| 4. Fusion with Group Now |  |  |  | .10  (*N*=240) | .01  (*N=*213) | .16^**^  (*N=*253) | .14^*^  (*N*=231) | .39^***^  (*N*=217) | .30^***^  (*N*=214) | .42^***^  (*N*=237) | .27^***^  (*N*=230) | .09  (*N*=225) |
| 5. Fusion with Family |  |  |  |  | .61^***^  (*N=*220) | .02  (*N*=236) | -.21^**^  (*N*=213) | -.07  (*N*=199) | .07  (*N*=196) | -.04  (*N*=219) | -.14^*^  (*N*=212) | -.21^**^  (*N*=207) |
| 6. Fusion with Spain’s National Justice System |  |  |  |  |  | -.21^**^  (*N*=210) | -.43^***^  (*N*=189) | -.17^*^  (*N*=177) | -.16^*^  (*N*=175) | -.15^*^  (*N*=237) | -.28^***^  (*N*=231) | -.27^***^  (*N*=236) |
| 7. Injustice |  |  |  |  |  |  | .42^***^  (*N*=234) | .22^***^  (*N*=221) | .30^***^  (*N*=219) | .19^***^  (*N*=242) | .15^*^  (*N*=235) | .10  (*N*=230) |
| 8. Personal Discrimination |  |  |  |  |  |  |  | .39^***^  (*N*=218) | .30^***^  (*N*=217) | .32^***^  (*N*=226) | .30^***^  (*N*=221) | .34^***^  (*N*=216) |
| 9. Sacrifices for Group |  |  |  |  |  |  |  |  | .63^***^  (*N*=213) | .42^***^  (*N*=215) | .34^***^  (*N*=212) | .25^***^  (*N*=207) |
| 10. Sacrifices for Value |  |  |  |  |  |  |  |  |  | .39^***^  (*N*=212) | .31^***^  (*N*=209) | .14^*^  (*N*=205) |
| 11. Shared values |  |  |  |  |  |  |  |  |  |  | .55^***^  (*N*=236) | .33^***^  (*N*=231) |
| 12. Shared positive experiences |  |  |  |  |  |  |  |  |  |  |  | .57^***^  (*N*=231) |
| 13. Shared negative experiences |  |  |  |  |  |  |  |  |  |  |  |  |

: ^*^*p* < .05, ^**^*p* < .01, ^***^*p* < .001.

**Table S19. Correlation between variables. Jihadists and non-Jihadist Muslims.**

| JIHADIST  NON-JIHADISTS | 1 | 2 | 3 | 4 | 5 | 6 | 7 | 8 | 9 | 10 | 11 | 12 | 13 |
| --- | --- | --- | --- | --- | --- | --- | --- | --- | --- | --- | --- | --- | --- |
| 1. Fusion with value at Time of Sentencing |  | .63^***^  (N=52) | .41^**^  (*N*=52) | .24  (*N*=51) | .09  (*N*=49) | -.30^*^  (*N*=45) | .21  (*N*=48) | .29^*^  (*N*=45) | .35^*^  (*N*=41) | .58^***^  (*N*=41) | .55^***^  (*N*=45) | .53^***^  (*N*=42) | .47^**^  (*N*=42) |
| 2. Fusion with Value Now | .67^***^  (*N*=86) |  | .46^***^  (*N*=52) | .54^***^  (*N*=52) | .35^*^  (*N*=50) | -.13  (*N*=46) | .28^+^  (*N*=49) | .33^*^  (*N*=46) | .26  (*N*=42) | .60^***^  (*N=*42) | .67^***^  (*N*=46) | .45^**^  (*N*=43) | .20  (*N*=43) |
| 3. Fusion with Group at Time of Sentencing | .42^***^  (*N*=85) | .37^***^  (*N=*85) |  | .67^***^  (*N*=55) | .15  (*N=*52) | -.04  (*N*=48) | .19  (*N*=52) | .39^**^  (*N*=46) | .33^*^  (*N*=42) | .40^**^  (*N*=41) | .58^***^  (*N*=48) | .46^**^  (*N*=45) | .18  (*N*=45) |
| 4. Fusion with Group Now | .37^***^  (*N=*85) | .44^***^  (*N*=85) | .68^***^  (*N*=90) |  | .08  (*N=*53) | .04  (*N=*49) | .29^*^  (*N*=52) | .50^***^  (*N=*46) | .24  (*N*=42) | .24  (*N*=41) | .49^***^  (*N*=48) | .40^**^  (*N*=45) | .28  (*N*=45) |
| 5. Fusion with Family | .17  (*N*=77) | .09  (*N*=77) | .16  (*N=*81) | .20  (*N*=82) |  | .47^***^  (N=49) | -.01  (*N*=49) | -.32^*^  (*N=*44) | -.06  (*N*=40) | .28  (*N*=39) | .24  (*N*=46) | -.02  (*N*=43) | .04  (*N*=43) |
| 6. Fusion with Spain’s National Justice System | .04  (*N=*76) | .02  (*N=*76) | .09  (*N=*80) | .18  (*N*=81) | .59^***^  (*N*=82) |  | -.42^**^  (*N*=45) | -.57^***^  (*N*=41) | -.33^*^  (*N*=38) | -.30  (*N*=38) | -.32^*^  (*N*=46) | -.46^**^  (*N*=44) | -.25  (*N*=44) |
| 7. Injustice | .31^**^  (*N=*79) | .08  (*N*=79) | .12  (*N*=83) | .09  (*N*=84) | .19  (*N*=77) | .07  (N=77) |  | .54^***^  (*N*=46) | .27  (*N*=42) | .44^**^  (*N*=41) | .27  (*N*=49) | .49^***^  (*N*=46) | .34^*^  (*N*=46) |
| 8. Personal Discrimination | .30^**^  (*N*=75) | .25^*^  (*N*=75) | .25^*^  (*N*=75) | .13  (*N*=75) | -.09  (*N*=77) | -.33^**^  (*N*=68) | .43^***^  (*N*=77) |  | .39^**^  (*N*=43) | .41^**^  (*N*=42) | .47^**^  (*N*=45) | .64^***^  (*N*=43) | .34^*^  (*N*=43) |
| 9. Sacrifices for Group | .16  (*N=*68) | .08  (*N=*68) | .42^***^  (*N=*67) | .20  (*N*=67) | .06  (*N*=60) | -.11  (*N*=60) | .24^+^  (*N*=69) | .43^***^  (*N*=67) |  | .66^***^  (*N*=40) | .35^*^  (*N*=42) | .46^**^  (*N*=41) | .43^**^  (*N*=41) |
| 10. Sacrifices for Value | .26^*^  (*N*=68) | .17  (*N*=68) | .10  (*N=*67) | .03  (*N*=67) | .10  (*N*=60) | -.08  (N=60) | .02  (*N=*70) | .06  (*N*=69) | .21  (*N*=67) |  | .52^**^  (*N*=40) | .59^***^  (*N*=39) | .38^*^  (*N*=39) |
| 11. Shared values | .49^**^  (*N*=68) | .56^***^  (*N*=69) | .41^***^  (*N*=73) | .48^***^  (*N*=74) | .13  (*N*=67) | -.05  (*N*=74) | .16  (*N*=76) | .18  (*N*=70) | .39^**^  (*N*=63) | .15  (*N*=64) |  | .57^***^  (*N*=46) | .30^*^  (*N*=46) |
| 12. Shared positive experiences | .37^**^  (*N*=65) | .30^*^  (*N*=65) | .47^***^  (*N*=69) | .48^***^  (*N*=70) | .15  (*N*=63) | -.04  (*N*=74) | .14  (*N*=72) | .12  (*N*=67) | .33^*^  (*N*=61) | .29^*^  (*N*=62) | .56^***^  (*N*=72) |  | .44^**^  (*N*=46) |
| 13. Shared negative experiences | .19  (*N*=61) | .21  (*N*=61) | .34^**^  (*N*=65) | .34^***^  (*N*=66) | .00  (*N*=59) | -.11  (*N*=66) | .01  (*N*=68) | .14  (*N*=63) | .23  (*N*=57) | .15  (*N*=59) | .44^***^  (*N*=68) | .43^***^  (*N*=68) |  |
| Notes: Correlations for jihadists above the Table, and for non-jihadists, below the Table. ^*^*p* < .05, ^**^*p* < .01, ^***^*p* < .001. | | | | | | | | | | | | | |

**Table S20. Correlation between variables. Latino gangs and Delinquent bands.**

| LATINO-GANGS.  DEL. BANDS | 1 | 2 | 3 | 4 | 5 | 6 | 7 | 8 | 9 | 10 | 11 | 12 | 13 |
| --- | --- | --- | --- | --- | --- | --- | --- | --- | --- | --- | --- | --- | --- |
| 1. Fusion with value at Time of Sentencing |  | .40^***^  (*N*=72) | .32^**^  (*N=*71) | .25^*^  (*N*=72) | -.18  (*N*=74) | -.15  (*N*=54) | -.04  (*N*=74) | .20  (*N=*69) | .36^**^  (*N*=67) | .30^*^  (*N*=67) | .30^*^  (*N*=72) | .35^**^  (*N*=72) | .22  (*N*=71) |
| 2. Fusion with Value Now | .37^*^  (*N*=40) |  | .25^*^  (*N=*69) | .68^***^  (*N*=70) | -.25^*^  (*N*=71) | -.22  (*N*=52) | .22  (*N*=71) | .25^*^  (*N*=67) | .61^***^  (*N*=66) | .59^***^  (*N*=67) | .42^**^  (*N*=70) | .38^**^  (*N*=70) | .14  (*N*=69) |
| 3. Fusion with Group at Time of Sentencing | .34^***^  (*N*=41) | .06  (*N*=40) |  | .25^*^  (*N*=72) | -.14  (*N*=75) | -.10  (*N*=55) | -.14  (*N*=74) | .24^+^  (*N=*66) | .33^**^  (*N*=65) | .26^*^  (*N*=65) | .37^**^  (*N*=71) | .34^**^  (*N*=71) | .37^**^  (*N*=70) |
| 4. Fusion with Group Now | .29  (*N*=41) | .65^***^  (*N*=40) | .40^**^  (*N*=45) |  | -.19  (*N=*73) | -.14  (*N*=52) | .15  (*N*=72) | .27^*^  (*N=*67) | .61^***^  (*N*=66) | .44^***^  (*N*=66) | .34^**^  (*N*=72) | .38^**^  (*N*=72) | .14  (*N*=71) |
| 5. Fusion with Family | .03  (*N=*30) | .04  (*N*=29) | -.15  (*N=*32) | -.04  (N=32) |  | .69^***^  (*N*=57) | -.09  (*N*=77) | -.26^*^  (*N*=69) | -.22  (*N*=67) | -.13  (*N*=67) | -.26^*^  (*N*=73) | -.23^+^  (*N*=73) | .23^+^  (*N*=72) |
| 6. Fusion with Spain’s National Justice System | .01  (*N*=29) | -.25  (*N*=28) | -.11  (*N=*31) | -.17  (*N*=31) | .67^***^  (*N*=32) |  | -.41^**^  (*N*=56) | -.40^**^  (*N*=49) | -.15  (*N*=48) | -.33^*^  (*N*=48) | -.14  (*N*=72) | -.10  (*N*=72) | -.18  (*N*=71) |
| 7. Injustice | -.13  (*N*=43) | .19  (*N*=42) | -.03  (*N=*45) | .09  (*N*=45) | -.40^*^  (*N=*33) | -.33  (*N*=32) |  | .29^*^  (*N=*68) | .13  (*N*=66) | .32^**^  (*N=*66) | .11  (*N*=72) | -.04  (*N*=72) | .03  (*N*=71) |
| 8. Personal Discrimination | -.09  (*N*=40) | .03  (*N*=39) | -.05  (*N*=43) | -.16  (*N*=43) | -.35^*^  (*N*=32) | -.42^*^  (*N*=31) | .34^*^  (*N*=44) |  | .49^***^  (*N*=65) | .43^***^  (*N=*65) | .33^**^  (*N*=68) | .20  (*N*=68) | .41^**^  (*N*=67) |
| 9. Sacrifices for Group | .21  (*N*=41) | .48^**^  (*N*=40) | .32^*^  (*N*=42) | .76^***^  (*N*=42) | .10  (*N*=32) | .05  (*N=*31) | --01  (*N=*44) | -.19  (*N*=43) |  | .72^***^  (N=64) | .32^**^  (*N*=67) | .28^*^  (*N*=67) | .16  (*N*=66) |
| 10. Sacrifices for Value | .20  (*N*=40) | .66^***^  (*N*=39) | .28  (*N*=40) | .75^***^  (*N*=40) | .24  (*N*=30) | .04  (*N*=29) | .16  (*N*=42) | .01  (*N*=41) | .78^***^  (*N*=42) |  | .29^*^  (*N*=67) | .17  (*N*=67) | .01  (*N*=67) |
| 11. Shared values | .28  (*N*=41) | .53^***^  (*N*=40) | .61^***^  (*N*=43) | .58^***^  (*N*=43) | .07  (*N*=33) | -.08  (*N*=45) | .17  (*N*=45) | .02  (*N*=43) | .57^***^  (*N*=43) | .63^***^  (*N*=41) |  | .50^***^  (*N*=73) | .29^*^  (*N*=72) |
| 12. Shared positive experiences | .21  (*N*=41) | .43^**^  (*N*=40) | .44^**^  (*N*=43) | .48^**^  (*N*=43) | .05  (*N*=33) | -.29^*^  (*N*=45) | .17  (*N*=45) | -.07  (*N*=43) | .30^*^  (*N*=43) | .37^*^  (*N*=41) | .56^***^  (*N*=45) |  | .51^***^  (*N*=72) |
| 13. Shared negative experiences | -.01  (*N*=41) | .26  (*N*=40) | .24  (*N*=43) | .35^*^  (*N*=43) | -.18  (*N*=33) | -.18  (*N*=46) | .17  (*N*=45) | .15  (*N*=43) | .12  (*N*=43) | .24  (*N*=41) | .22  (*N*=45) | .47^**^  (*N*=45) |  |
| Notes: Correlations for Latino gangs above the Table, and for delinquent bands, below the Table. ^*^*p* < .05, ^**^*p* < .01, ^***^*p* < .001. | | | | | | | | | | | | | |

**Table S21. Univariate Analyses for each of the outcome measures on each group (*F*s, Means and Standard Deviations).**

**Other Supplementary Materials for this manuscript include the following documents on Human Subjects Protection:**

Concurrence

Dr. Ángel Gómez was given permission by Spain's Ministry of the Interior to conduct psychological studies in Spanish prisons of internees under the following conditions of informed consent: 1. participants must be informed of the purpose and procedure of the study using measures previously tested in non-prison populations and that have never before caused discomfort or harm, 2. participation must be entirely voluntary, 3. all data must be anonymized, 4. participants can end participation at any time for any thematic indicator. These prison studies were designed to test theories and measures used and developed for previous studies of non-prison populations that were done in collaboration with Artis researchers under IRB approval (pages 51-53 include the permission of the Ministry of Interior).

Dr. Gómez informed Artis of the initiation of the prison studies under Spanish Ministry approval (Wave 1), and of the possibility of follow up studies (wave 2), and inquired if Artis researchers could assist in analyzing the data once all of the studies were complete.

Artis IRB chair reviewed the conditions on the study detailed by the Spanish Ministry of the Interior and authorized a letter of concurrence stating that Artis would call a full meeting of its IRB once all the prison studies were completed with the purpose of then deciding if the studies complied with Artis IRB requirements for assisting Dr. Gómez's team in the analysis of data from those studies (pages 54-56 include the IRB documents, in particular, a first document, and an extension).

A second letter of concurrence to the same effect was provided by the Artis IRB chair after being informed by Dr. Gómez that additional studies (Wave 2) would be initiated under Spanish Ministry approval. The Universidad de Educación a Distancia in Spain granted final approval to proceed with analyses after reviewing materials meeting all requirements for human subjects approval (included in page 57):
